# Dissection of innate-immune-ligand- and interferon-protein-mediated transcriptional responses in human THP1 cell states

**DOI:** 10.1038/s42003-025-09343-7

**Published:** 2026-02-12

**Authors:** Lodoe Lama, Pavel Morozov, Aitor Garzia, Thomas Tuschl

**Affiliations:** Laboratory for RNA Molecular Biology, The Rockefeller University, New York, NY USA

**Keywords:** Pattern recognition receptors, Gene regulation in immune cells

## Abstract

Interferon regulatory factors (IRFs) are essential for transcription of interferons (IFNs), interferon-stimulated genes (ISGs), and pro-inflammatory cytokines. We profile the transcriptome of human monocyte THP1 cells challenged with cGAMP, LPS, or IFNB1 protein as a function of knockout (KO) or overexpression (OE) of IRFs or KO of IFNAR2. We define distinct gene expression groups, reflecting the transcription factors responsible for their induction including subgroups activated by more than one pathway or feed-forward regulation. We compare IRF3- and IRF7-induced gene signatures and note the strong direct induction of a subset of antiviral-acting ISGs by IRF3 or IRF7. LPS treatment induces NF-κB responses in monocyte and macrophage state cells, however, IFNs and ISGs are only co-induced in the macrophage state requiring IRF3. IRF1, IRF2, IRF5, and IRF8 are largely dispensable for IFN-stimulated or innate-immune-mediated gene induction. This study provides a valuable resource for dissecting complex inflammatory gene signatures and their underlying transcription factors thereby anticipating the effects of selectively drugging the underlying pathways.

## Abstract (web summary)

Transcriptomic profiling of THP1 cells challenged with cGAMP, LPS, or IFNB1 protein under IRF knockout or overexpression conditions, or IFNAR2 knockout, reveals distinct IRF3- and IRF7-driven antiviral programs and pathway-selective gene signatures.

## Introduction

Interferons (IFNs), chemokines, and pro-inflammatory cytokines are secreted signaling proteins central to innate and adaptive immunity for defense against pathogens^1^. These signaling proteins are induced upon activation of pattern recognition receptors (PRRs) for pathogen-associated molecular patterns (PAMPs)^2^ and damage-associated molecular patterns (DAMPs)^3^ of compromised host cell. PRRs include toll-like receptors (TLRs)^4^, retinoic-acid-inducible-gene-I- (RIG-I-) like-receptors (RLRs)^5^, and cyclic GMP-AMP synthase (CGAS)^6^. These PRRs, when activated, engage various adaptor proteins and kinases leading to phosphorylation and activation of members of various families of transcription factors including interferon regulatory factors (IRFs) and NF-κB complexes^7^. The expressed IFN and cytokine proteins engage in autocrine and paracrine signaling triggering additional transcriptional responses, including the production of hundreds of IFN-stimulated genes (ISGs), thereby complicating deconvolution of the initiating inflammatory response. Cells exposed only to type I IFN protein do not transcribe interferon genes. A feed-forward induction of type I IFN genes requires long-lasting or repeated innate immune stimulation ensuing IFN protein synthesis^8,9^.

IFNs and ISGs lead into a cellular antiviral state suppressing intracellular pathogen replication. Co-induced chemokines and pro-inflammatory cytokines alert the adaptive pathogen-selective immune system to initiate T and B cell development for clearance of infected cells and prevention of new infection, respectively^10^.

IFNs are categorized into three types based on the cell surface receptors they engage^1^. Relevant to innate responses are type I and type III IFN genes^11^. Type I includes IFNβ (*IFNB1*), IFNε (*IFNE*), IFNκ (*IFNK*), IFNω (*IFNW*), and 13 IFNα (*IFNA1*, *2*, etc.) and type III includes IFNλ1 (*IFNL1*), IFNλ2 (*IFNL2*), and IFNλ3 (*IFNL3*). Type I IFNs bind the IFNAR1-IFNAR2 cell-surface-localized heterodimeric receptor while type III IFNs bind IFNLR1-IL10RB. IFNAR1 and IFNAR2 are broadly expressed while IFNLR1 and IL10RB are enriched in epithelial and immune cells, respectively. Engagement of type I or type III IFN receptors activates JAK1 and TYK2 kinases which in turn phosphorylate STAT1 and STAT2 transcription factors. Phosphorylated STATs form various transcription factor complexes^12,13^ responsible for induction of ISGs.

Concomitant with IFNs is the induction of CXCL (CXCL10, CXCL11)^14^ and CCL (CCL3, CCL4, CCL5) family chemokines^15,16^. These chemokines mediate their effects by binding to their respective CXCR or CCR receptors present on target cells, initiating diverse intracellular signaling cascades^17^. Activation of the NF-κB pathway leads to the production of pro-inflammatory cytokines (IL1B, IL8, TNF)^18^, a subset of which needs proteolytic processing for their activation and pyroptosis for their release and intercellular signaling^19^.

The human genome encodes nine members of the IRF family, defined by an N-terminal DNA-binding domain that recognizes GAAA dsDNA sequence motifs^20^. IRF1, 3, 5, 7, and 8 have been implicated in IFN expression. IRF1 was the first member identified^21^ though subsequent studies in IRF1 knockout (KO) mice^22^ implicated IRF3 and IRF7 as major IFN transcription factors active upon viral infection^23^. IRF3 expression is constitutive, while *IRF1*, *IRF7*, and *IRF9* are also ISGs, IRF1 and IRF7 being responsible for feed-forward IFN gene transcription during persistent innate immune activation^24–26^. IRF3 and IRF7 vary in dsDNA-binding specificity, with IRF7 being able to induce expression of all type I IFN genes, while IRF3 is selective for IFNB1^25^. It should be noted that plasmacytoid dendritic cells (pDCs), which are known for their ability of massive IFN production, show constitutively high expression of IRF7^27^. IRF5 is implicated in TLR7- and TLR8-dependent IFN induction^28,29^. IRF8, which is predominantly expressed in human blood monocytes, constitutively binds to the IFNB1 promoter and facilitates rapid IRF3-dependent transcription of IFNB1 upon viral infection^30^. IRF9 participates in downstream IFN-receptor-mediated activation of ISGs by forming the phosphorylated STAT1-STAT2-IRF9 ISGF3 complex^13^.

The subcellular localization of the PRRs is also regulated and differentially impacts the various inflammatory pathways. In monocytes, TLR4 is predominantly located on the plasma membrane^31^ and mostly signals via the TIRAP-MYD88 adapter complex^32^ leading to downstream activation of NF-κB pathway. As shown in bone-marrow-derived macrophages, the selectively co-expressed GPI-anchored protein CD14^33^ facilitates LPS-induced TLR4 endocytosis and engagement of the TRAM-TRIF pairs of adapter proteins^34^ leading to co-induction of IRF3-dependent IFN and ISG expression aside from the TIRAP-MYD88-mediated NF-κB gene activation^35^.

The role of IRFs as transcriptional regulators of innate immune response has been established, particularly for the induction of IFNs and ISGs^36^. However, a recent review^37^ underscores a considerably more complex innate immune signaling processes extending beyond a sequential transcriptional response for IRF-induced IFNs and IFN-protein-induced ISGs. For instance, IRF3 had been implicated in direct upregulation of the prototypical antiviral-acting ISGs *IFIT1*, *2*, *3*, and *ISG15* independent of IFN receptor signaling^38^.

The human leukemia-derived monocyte THP1^39^ cell line is widely used in immune modulation studies^40^ encompassing inflammatory responses^41^, host-pathogen interaction mechanisms^42–44^, phagocytosis and antigen presentation mechanisms^45^, toxicology in drug development^46^, and macrophage polarization^47^. Treatment of THP1 monocytes with phorbol 12-myristate 13-acetate (PMA), a mimic of the second messenger diacylglycerol, leads to their terminal differentiation into adherent macrophage-like cells of the M0 state with remarkable phenotypic and functional characteristics of human monocyte-derived macrophages derived from peripheral blood mononuclear cell^48,49^. M0 macrophages can be further polarized into M1- or M2-type “activated” macrophages by treatment with IFNγ-LPS or interleukin-4 (IL-4), respectively^47^. Given that genes involved in innate immunity are non-essential in cultured THP1 cells, their roles are readily explored using CRISPR-based gene KO. Individual studies in THP1 cells utilizing KO of various PRR genes^50,51^, IRF3^52^, IFNAR receptor^53^, and IRF9^54^ have helped to establish and validate their roles in IFNs and ISG induction. However, a comprehensive study dissecting transcriptional activation by individual IRF family members in innate immune sensing and downstream IFN-protein-mediated induction of ISGs and other inflammatory pathways is still lacking. Notably, only a single study compared the role of six IRF KOs in human alveolar epithelial A549 cell line by monitoring a small subset of IFN, cytokine, and ISG mRNAs by RT-qPCR^55^.

Here we characterized THP1 wild-type and KO cells by transcriptome-wide RNA-seq to classify innate immune responses and their transcriptional pathways as a function of IRFs and IFNAR2. We defined 1) CGAS-STING1-induced genes dependent on IRF3 or IRF7, including type I IFNs, 2) a subset of ISGs that are also directly induced by IRF3 or IRF7, 3) IRF9-dependent and independent ISGs, and 4) NF-κB-pathway-induced genes. Activation of the CGAS-STING1 pathway using cGAMP does not activate the NF-κB pathway. However, LPS treatment activated the NF-κB response in monocyte and macrophage THP1 cell states, and selective co-induction of type I IFNs and ISGs in macrophages only.

## Results

### THP1 cells as model system to dissect innate immunity transcriptional responses

THP1 cells expressed PRRs *CGAS*, *DDX58/RIG-I*, *IFIH1/MDA5*, *TLR2*, and *TLR4* (Supplementary Data 1, Supplementary Fig. 1A). They also expressed intermediary adapters *STING1*, *MAVS*, *MYD88*, *TIRAP*, *TRAM*, *TRIF*, kinases *TBK1*, *IKBKB*, *IKBKE*, and *CHUK*, and seven of the nine *IRFs* and *NF-κB* transcription factors essential for PRR-mediated transcriptional response including activation of IFN genes. Additionally, THP1 cells expressed *IFNAR1* and *2*, *TYK2*, *STAT1*, *STAT2*, and *IRF9* required for induction of ISGs. The *IFNLR1* receptor required for type III IFN-mediated induction of ISGs was not expressed. *CD14* required for internalization of TLR4^33^ was only expressed in macrophage THP1 cells.

A time course of cGAMP treatment using digitonin-permeabilized THP1 monocytes revealed a biphasic posttranslational response (Supplementary Fig. 1B). The first phase of phosphorylation of STING1, TBK1, and IRF3 S386 was detectable at 0.5 h, plateaued at 2.5 h, and persisted up to 4.5 h post treatment. The second phase, marked by phosphorylation of STAT1 Y701, emerged 2.5 h post treatment. By contrast, direct IFNB1 protein treatment of THP1 cells showed a less than 30 min onset of STAT1 Y701 phosphorylation and lasted for about 4 h (Supplementary Fig. 1C). The first phase following cGAMP exposure corresponded to the innate immune sensing as exemplified by IRF3-dependent IFNB1 protein synthesis, the second phase was caused by IFNAR signaling due to IFNB1 protein binding. The 2-h delay in STAT1 Y701 phosphorylation by cGAMP treatment versus direct IFNB1 protein exposure represents the time needed for IFNB1 gene transcription and translation. Similarly, increased protein levels of ISGs, such as IRF9, were only noticeable 3 h after IFNB1 protein treatment, and further increased and lasted for at least 8 h.

*IFNB1* and *IFIT1* mRNAs were induced 1 h post treatment (Supplementary Fig. 1D) but increased further and plateaued at 2 h. *IFIT1*, but not *IFNB1*, was also induced 2 h post IFNB1 protein treatment (Supplementary Fig. 1E). Therefore, *IFIT1* is a direct target of innate immune activation as well as an ISG^38^. A 2 h time point after cGAMP delivery by lipid-nanoparticle-based transfection or digitonin-mediated membrane permeabilization showed 24- or 260-fold increased *IFNB1* mRNA abundance by RT-qPCR, respectively, compared to plain addition of cGAMP into the culture medium (Supplementary Fig. 1F). We selected digitonin-mediated cGAMP delivery for this study because it showed the highest level of *IFNB1* mRNA induction.

LPS treatment of monocyte THP1 cells led to selective activation of the NF-κB pathway 1 h post treatment as monitored by RELA phosphorylation in absence of any IRF3 phosphorylation and at concentrations of up to 1 µg/ml LPS in culture medium (Supplementary Fig. 1G). However, LPS treatment of macrophage THP1 cells additionally induced *IFNB1* mRNA (Supplementary Fig. 1H).

In summary, these time-dependent observations prompted us to select a 2 h time point for RNA-seq analysis of the innate immune responses. This time point captured the peak of early and direct transcriptional responses by innate immune sensing exemplified by *IFNB1* mRNA production. A 2 h time point following innate immune activation also captured the onset of interferon-protein-stimulated gene (ISG) transcription but was still ahead of ISG protein synthesis and therefore free of feed-forward gene regulation. A complete set of ISGs was determined by direct treatment of cells with IFNB1 protein for 2 and 24 h^56,57^. Finally, we included a subset of LPS-treated macrophage THP1 cells to capture the CD14-dependent TLR4-mediated co-induction of IFNs and ISGs.

### Comparative transcriptomic analysis identified IRF-, STAT-, NF-κB-, and stress-response-specific gene sets in THP1 monocytes

To assess the individual contributions of IRF family members to innate immunity transcriptional responses, we created KO lines for IRF1, 2, 3, 5, 7, 8, and 9 including the double KO (DKO) of IRF3 and 7 (Supplementary Figs. 2, 3). We used lentiviral expression of various IRFs to confirm rescue of KO lines. We also generated an IFNAR2 KO line to unmask IRF-induced transcription from IFNB1 protein signaling and ISG expression.

**Fig. 1:**
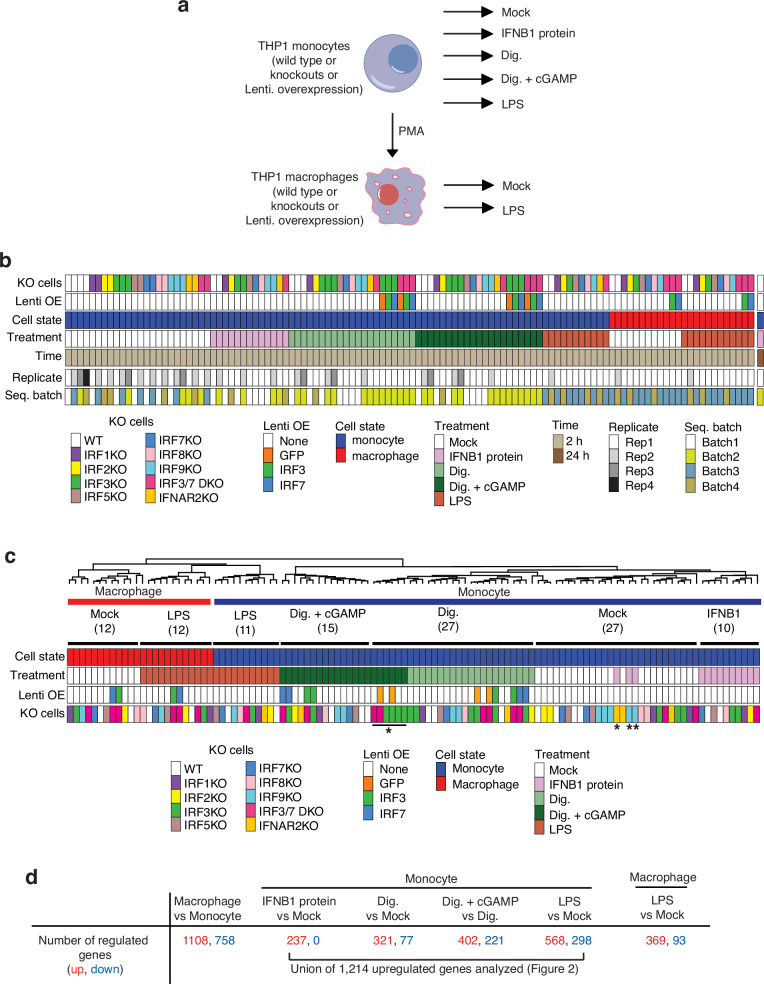
Summary of experimental approach and analysis. **a** THP1 cell states and treatments. **b** Sample overview for transcriptome analysis. **c** Organization of samples into seven subgroups by unsupervised clustering as shown in Supplementary Fig. 4. Numbers in parentheses denote the total number of samples included in the DGE analysis for each subgroup. * denotes outlier samples that did not cluster with their expected treatment group. **d** Differentially regulated gene from comparing responses to the various treatments. A total of 1214 upregulated genes in monocytes under various treatments were selected for further analysis as shown in Fig. 2. Dig. digitonin, Lenti OE lentiviral OE of the indicated protein.

We exposed wild-type and KO THP1 cells to different innate immune stimuli or IFNB1 protein (Fig. 1a) and performed polyA RNA-seq yielding 114 expression profiles (Fig. 1b, Supplementary Data 2). For RNA-seq data analysis, we first conducted pairwise comparisons to identify genes which were ≥4-fold or ≥8-fold regulated by digitonin treatment, immune stimulation or differentiation, respectively, and expressed ≥300 TPM in the induced state, resulting in 360 genes. This gene set was used for unsupervised clustering of all samples and yielded seven sample subgroups distinguished by cell state and treatment conditions (Fig. 1c, Supplementary Fig. 4). Isogenic IRF and IFNAR2 KO cell lines clustered with wild-type THP1 cells unless the treatment response depended on the KO gene. For instance, cGAMP-treated IRF3 KO and IRF3/7 DKO samples clustered together with mock-digitonin-treated sample subgroup and IFNB1-protein-treated IFNAR2 KO sample clustered with the group of untreated samples. We therefore considered RNA profiles of isogenic lines that clustered into sample subgroups equivalent to biological replicates in subsequent differential gene expression (DGE) analysis.

The seven sample subgroups underwent group-wise DGE analyses to identify regulated genes with statistical significance (adjusted *p*-value < 0.05, Supplementary Data 3, Supplementary Fig. 5). This included comparisons between monocyte and macrophage cell states, and between cell-state-specific mock and treated conditions. We identified 3039 significantly regulated genes (≥2-fold) and average transcript abundance >20 TPM in the induced state (Fig. 1d). Monocyte-to-macrophage terminal differentiation resulted in 1108 up- and 758 downregulated genes, indicating extensive transcriptional reprogramming. IFNB1 protein, digitonin, cGAMP, or LPS treatment resulted in 1214 up- and 560 downregulated genes in monocytes, some of which were regulated by more than one treatment. LPS treatment of macrophages yielded 369 up- and 93 downregulated genes, of which 237 and 26 were also up- and downregulated, respectively, by LPS treatment of monocytes. Given the short 2 h time points and the rapid transcriptional activation driving innate immunity responses, we did not explore downregulated genes. Also, we did not pursue the small subset of genes uniquely regulated in LPS-treated macrophages.

**Fig. 2:**
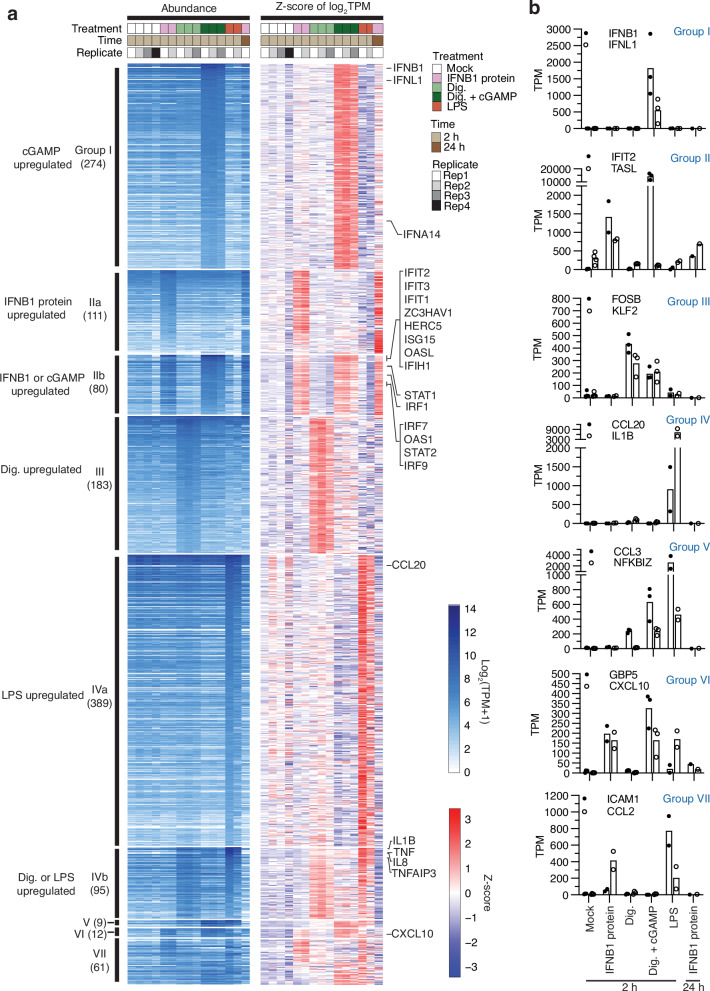
Gene groups upregulated by different treatments in THP1 monocytes. **a** Heatmaps displaying either absolute abundance (TPM, left panel) or row-normalized differential expression (z-score, right panel) of upregulated genes under various treatments in parental THP1 monocytes. Genes are grouped based on their unique or overlapping regulation across treatments as labeled on the left side of the heatmap. Genes assigned to various groups are listed in Supplementary Data 3. Each row corresponds to a gene, while each column represents a sample, either untreated or treated with IFNB1 protein, digitonin, digitonin with cGAMP, or LPS. Genes within each group are sorted by decreasing abundance. Numbers in parentheses denote the total number of genes identified per group. Representative genes are indicated by name. The transcript abundance scale also applies to subsequent figures. Dig. digitonin, TPM transcripts per million. **b** Expression levels (TPM) of representative genes from each gene group in parental THP1 monocytes. Each data point represents the TPM value for an individual sample, as listed in Supplementary Data 3, with the mean indicated by the bar.

The induced genes in monocytes were further subdivided by whether they were uniquely regulated by one or more than one of the treatments, resulting in seven groups of genes (Fig. 2, Supplementary Data 3). Unsupervised clustering of samples and averaged z-scores of these gene groups organized samples according to treatment, unless their treatment response was impacted by KO of IRF or IFNAR2 (Supplementary Fig. 6A). The outliers were cGAMP-treated IRF3 KO and IRF3/7 DKO, which clustered with mock-treated controls, and IFNB1-protein-treated IFNAR2 KO, which clustered with untreated samples. These gene groups represent IRF-, STAT-, stress-, and NF-κB-dependent gene regulation or a combination thereof.

### Group I genes induced by IRF3 and IRF7 following cGAMP treatment

**Fig. 3:**
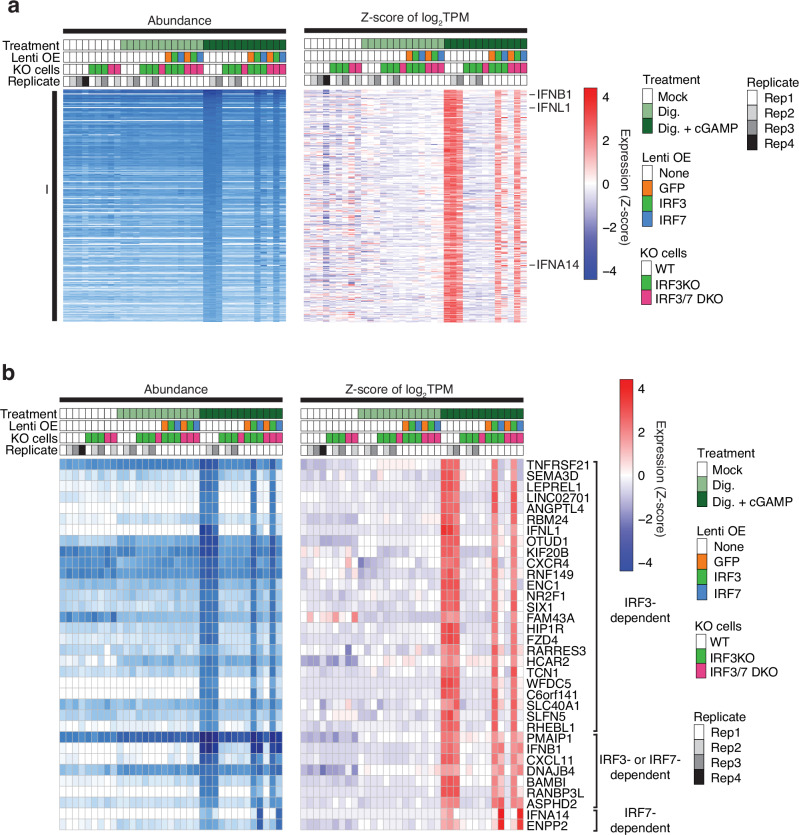
Validation of Group I genes using IRF KO and OE in THP1 monocytes. **a** Group I genes uniquely induced by cGAMP in wild-type THP1 are impacted by IRF KO or OE. **b** A selected subset of Group I genes demonstrating distinct IRF3- or IRF7-dependent induction in response to cGAMP treatment. A total of 88 Group I genes (log_2_FC > 2, TPM > 20) were evaluated, and those showing the following fold induction threshold criteria in OE samples are shown: IRF3-dependent (log_2_FC > 2 in IRF3 OE, log_2_FC < 2 in IRF7 OE), IRF3- or IRF7-dependent (log_2_FC > 2 in IRF3 or IRF7 OE), and IRF7-dependent (log_2_FC > 2 in IRF7 OE, log_2_FC < 2 in IRF3 OE). Genes uniquely or commonly upregulated by IRF3 or IRF7 are indicated. Only 25 of the 45 identified IRF3-dependent genes are arbitrarily selected and displayed for simplicity. Dig. digitonin, Lenti OE lentiviral OE of the indicated protein.

DGE comparing cGAMP- versus mock-digitonin-treated sample subgroups (Fig. 1c) identified 402 genes that were upregulated ≥2-fold. A subset of 274 of these genes, referred to as Group I, was exclusively upregulated in cGAMP-treated samples and showed no regulation in IFNB1-protein- or LPS-treated versus control subgroups (Fig. 2). Group I genes included highly induced *IFNB1* and *IFNL1* (≥1000 TPM) as well as *SEMA3D*, *LEPREL1*, and *SIX1* (≥100 TPM), a set of genes with potential roles in innate immune responses (Fig. 3).

IRF3 KO or IRF3/7 DKO monocytes did not respond to cGAMP (Fig. 3a). Stable ectopic overexpression (OE) of IRF3 in IRF3 KO or IRF3/7 DKO cells restored cGAMP-dependent induction of most Group I genes, indicating that these were direct targets of IRF3. The transcript abundance of *IRF7* was 3-fold less compared to *IRF3*, and IRF7 protein abundance was too low (Supplementary Fig. 6B, C) to induce Group I genes by cGAMP in IRF3 KO monocytes. IRF7 OE in IRF3 KO or IRF3/7 DKO monocytes induced 7 (10%) out of 88 Group I genes (≥4-fold) by cGAMP, including *IFNB1* or *CXCL11*, and *IFNA14* and *ENPP2*, which were not rescued by IRF3 OE (Fig. 3b, Supplementary Fig. 7A, B). IFNB1 protein-pretreated cells exposed to cGAMP induced all Group I genes including IRF7-selective *IFNA14* and *ENPP2* (Supplementary Fig. 7C). *IRF7* is an ISG and was 6-fold increased by pre-treatment of wild-type monocytes with IFNB1 protein. IFNB1 protein-pretreatment applied to IRF3 KO cells and followed by cGAMP treatment was insufficient to induce *IFNA14* or *ENPP2*. The induced *IRF7* mRNA abundance was 3-fold less compared to IRF7 OE in IRF3 KO cells. Together, these experiments suggest that IRF3 and IRF7 act cooperatively in Group I gene induction, perhaps as phosphorylated IRF3-IRF7 heterodimers^58^.

We also knocked out IRF1, IRF2, IRF5, IRF7, IRF8, IRF9, or IFNAR2. Except for IRF8, gene expression of untreated cell states was comparable to wild-type. IRF8 is a transcription factor implicated in myeloid lineage specification^59,60^. *IRF8* was 6-fold more abundant compared to *IRF3* in wild-type monocytes (Supplementary Fig. 6B). IRF8 KO resulted in 5-fold upregulation of the protease *CTSG* compared to wild-type, and it became one of the most abundant transcripts in monocytes. However, comparing wild-type THP1 monocytes to macrophages, we observed a simultaneous 10-fold and 8-fold reduction of *CTSG* and *IRF8*, respectively. Comparing IRF8 KO to wild-type macrophage, *CTSG* was still 10-fold increased, implying IRF8 as a repressor of *CTSG* in either cell state. With respect to 2 h cGAMP treatment, KO of neither IRF1, IRF2, IRF5, IRF7, IRF8, IRF9, or IFNAR2 impaired Group I gene induction (Supplementary Fig. 8). The mRNA abundance of *IRF1* and *IRF2* was comparable to *IRF7*, and therefore possibly too low to impact Group I expression, while *IRF5*, *IRF9*, and *IFNAR2* were comparable to *IRF3* (Supplementary Fig. 6B).

### Group II genes induced by IFNB1 protein treatment (ISGs) also include a subset of IRF3- and IRF7-driven genes induced by cGAMP

DGE comparing 2 h IFNB1-protein- versus untreated sample subgroups (Fig. 1c) identified 173 genes that were upregulated ≥2-fold. Increasing the extracellular IFNB1 protein concentration 100-fold did not further increase the number or level of ISGs after 2 h treatment versus control (Supplementary Fig. 9A). Furthermore, we identified an additional 64 genes upregulated ≥4-fold after 24 h IFNB1 protein treatment versus control, representing ISGs with delayed induction kinetics, e.g. *IFI27* (Supplementary Fig. 5A). The combined 237 ISGs were then subdivided into groups depending on their induction by one or more treatment(s) (Fig. 2).

**Fig. 4:**
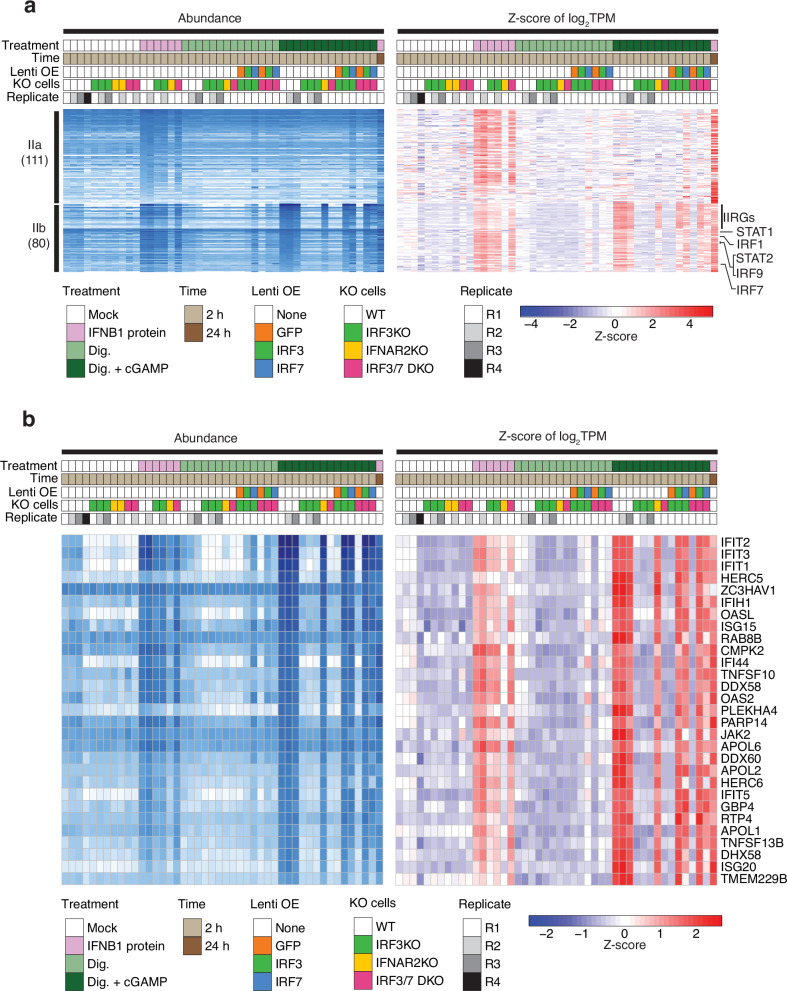
Validation of Group II (IIa and IIb) ISGs using IFNAR2 KO, IRF KO and OE in THP1 monocytes. **a** All Group II ISGs required IFNAR2 for induction by IFNB1 protein in the absence of cGAMP. Group IIb genes representing ISGs induced by IFNB1 protein or cGAMP in wild-type THP1 were impacted by IFNAR2 KO as well as IRF KO and OE. A subset of 29 Group IIb ISGs (IIRGs) also inducible by IRF3, retained strong induction (log_2_FC > 2) in IFNAR2 KO sample following cGAMP treatment and are marked with a black side bar. **b** IIRGs are shown in expanded view. IRF3 or IRF7 OE in KO samples rescued their induction following cGAMP treatment.

Group IIa comprises 111 ISGs uniquely induced in 2 h IFNB1-protein-treated cells, but still quiescent in 2 h cGAMP treatment (Fig. 2, Group IIa). Group IIb comprises 80 ISGs, which were also induced by 2 h cGAMP treatment. Group IIa and IIb required IFNAR2 for their induction by IFNB1 protein in absence of cGAMP (Fig. 4a).

Group IIb genes were either directly induced by phosphorylated IRF3 and IRF7 and/or represented the onset of autocrine IFNAR-dependent IFNB1 protein signaling. To differentiate between these possibilities, we treated IFNAR2 KO cells with cGAMP and continued to observe the induction of a subset of 29 Group IIb genes, including the antiviral ISGs *IFIT1*, *IFIT2*, *IFIT3*, *OASL*, and *ISG15* (Fig. 4b). We refer to these dual-regulated genes as IRF3/7-IFNAR-response genes or IIRGs. The IIRG induction in TPM was also up to 10-fold higher by cGAMP compared to IFNB1 protein treatment. Induction by cGAMP of Group IIb genes was abolished by IRF3 KO or IRF3/7 DKO and rescued by IRF3 or IRF7 OE. The remaining Group IIb genes are IFNAR-dependent rapidly induced ISGs and include all key regulators of feed-forward amplification, such as *IRF1*, *IRF7*, *IRF9*, *STAT1*, and *STAT2*.

KO of IRF1, IRF2, IRF5, IRF7, or IRF8 did not alter Group II gene induction by IFNB1 protein or cGAMP treatment (Supplementary Fig. 9B). The impact of IRF9 KO will be described in a separate section below.

### Group III stress response genes observed upon digitonin permeabilization for cGAMP delivery

Digitonin used for cellular permeabilization to facilitate cGAMP entry into cells caused cellular stress and resulted in upregulation of 321 genes, 183 of which were uniquely upregulated by digitonin (Fig. 2, Group III). Group III genes included membrane-localized annexin *ANXA1*, heat shock protein *HSP5A*, and transcription factors *FOSB*, *KLF2*, and *KLF6* (Supplementary Fig. 5B) and excluded interferons and ISGs (Supplementary Fig. 1D). None of the Group III genes were impacted by any IRF or IFNAR2 KO (Supplementary Fig. 10A).

### Group IV NF-κB-dependent genes induced by LPS

**Fig. 5:**
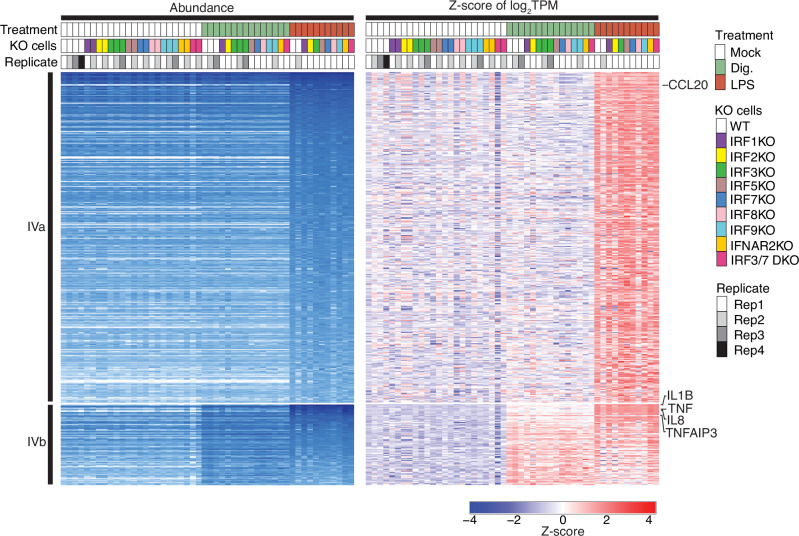
Group IVa and IVb NF-κB-dependent genes are unaffected by IRF or IFNAR2 KOs in THP1 monocytes. Group IVa genes exclusively induced by LPS and Group IVb genes primarily induced by LPS genes with minor co-induction by digitonin in wild-type and various KO THP1 monocytes. Induction of these genes remained unaffected by the KO of IRF or IFNAR2. Representative key pro-inflammatory genes are indicated by name.

LPS treatment yielded 568 upregulated genes (Fig. 1c, Supplementary Fig. 5D), of which 389 were only induced by LPS treatment (Fig. 2, Group IVa) while another 95 genes were also induced albeit to a lesser magnitude by digitonin treatment (Fig. 2, Group IVb). Group IVa and IVb comprise many classical NF-κB-dependent pro-inflammatory genes such as *CCL4*, *CCL20*, *IL1B*, *IL8*, *TNF*, and *TNFAIP3*, reconfirming LPS as strong TLR4-mediated activator of the NF-κB pathway in THP1 monocytes^15^. The induction of Group IV genes was not compromised by KO of IRF or IFNAR2 genes (Fig. 5).

### Group V genes induced by cGAMP or LPS

**Fig. 6:**
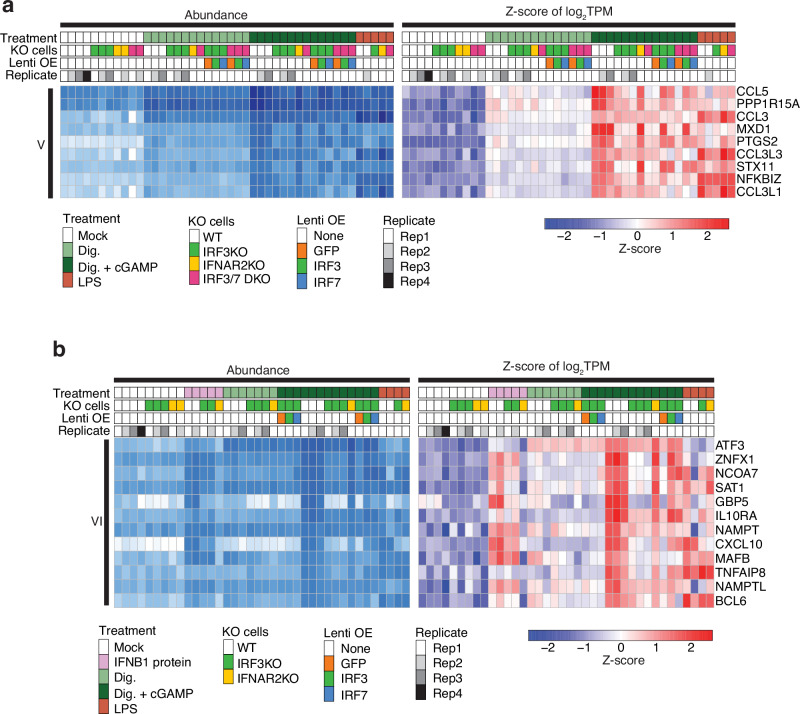
Validation of Group V and VI co-induced genes using IFNAR2 KO, IRF KO and OE in THP1 monocytes. **a** Group V genes co-induced by cGAMP or LPS in wild-type THP1 cells were impacted by IRF KO and OE. Their induction depended on IRF3 in cGAMP-treated samples but was IRF3-independent in LPS-treated samples. **b** Group VI genes co-induced by cGAMP, IFNB1 protein, or LPS in wild-type THP1 were impacted by IFNAR2 KO as well as IRF KO and OE. IFNAR2 was required for IFNB1-protein-induced activation but was dispensable in cGAMP-induced activation. IRF3 OE restored activation in response to cGAMP.

We identified 9 genes (Fig. 2, Group V) that were induced by cGAMP or LPS and nominally by digitonin. These genes were well expressed covering a range of 400–5500 TPM, including the secreted cytokine *CCL3* and *CCL5* and transcriptional regulator *NFKBIZ* (Fig. 6a). Group V gene induction required IRF3 but not IFNAR2 in cGAMP-treated samples. OE of IRF3 in IRF3 KO cells rescued their induction, indicating that these genes are direct targets of IRF3. Furthermore, LPS-mediated upregulation of these genes was independent of IRF3 or IFNAR2, suggesting these genes are also direct targets of NF-κB-mediated signaling.

### Group VI genes induced by cGAMP, IFNB1 protein, or LPS

A group of 12 genes (Fig. 2, Group VI) was induced by treatment with either cGAMP, IFNB1 protein, or LPS, indicating promoter or enhancer elements targetable by multiple transcription factors (Fig. 6b). This included the cytokine *CXCL10* and ISGs not fitting into Group II, e.g. *GBP5*. IFNB1-protein-induced activation of *CXCL10* required IFNAR2, but not IRF3. The induction following cGAMP treatment required IRF3 but not IFNAR2. Reconstitution of IRF3 or IRF7 in IRF3 KO cells restored its induction, indicating *CXCL10* is also direct target of IRF3 and IRF7. Finally, LPS-induced activation of this gene was nominally affected by the absence of IRF3 or IFNAR2, also indicating its NF-κB pathway-dependent regulation.

### Group VII genes with atypical induction profiles

A group of 61 genes (Fig. 2, Group VII) could not be confidently assigned to a particular group because of overlapping induction by various treatments and/or partial response to IRF or IFNAR2 KOs. This included 34 ISGs (e.g. *CCL2*, *ICAM1*, *GBP2*) that required IFNAR2 for induction following IFNB1 protein treatment. However, these ISGs also showed IFNAR2-independent induction by digitonin and/or LPS.

### ISGs induced by IFNB1 in absence of IRF9

KO of IRF9 severely blunted the IFNB1 protein response of most ISGs (Group II, VI, and VII). IRF9 and phosphorylated STAT1 and STAT2 are required for the induction of the majority of ISGs^13^. 39 (22%) out of 173 ISGs induced by 2 h IFNB1 protein treatment were impacted less than 1.5-fold by IRF9 KO (Supplementary Fig. 10B, Supplementary Data 4), including IRF1 (Supplementary Figs. 2A, D). To assess if these IRF9-independent ISGs were also STAT-dependent, we reevaluated RNA-seq data from STAT1 and STAT2 KO in HeLa cells obtained 16 h after IFNB1 protein treatment^61^. We found only 5 out of these 39 genes were induced in IFNB1-protein-treated wild-type HeLa cells (log_2_FC > 1, pAdj < 0.05, RPM > 20). These genes were no longer induced by IFNB1 protein treatment in STAT1 KO HeLa cells, while STAT2 KO cells showed a reduced induction compared to wild-type cells, consistent with the idea of STAT1 homodimers being responsible for their expression.

### Genes induced by LPS in PMA-differentiated macrophage THP1 cells

**Fig. 7:**
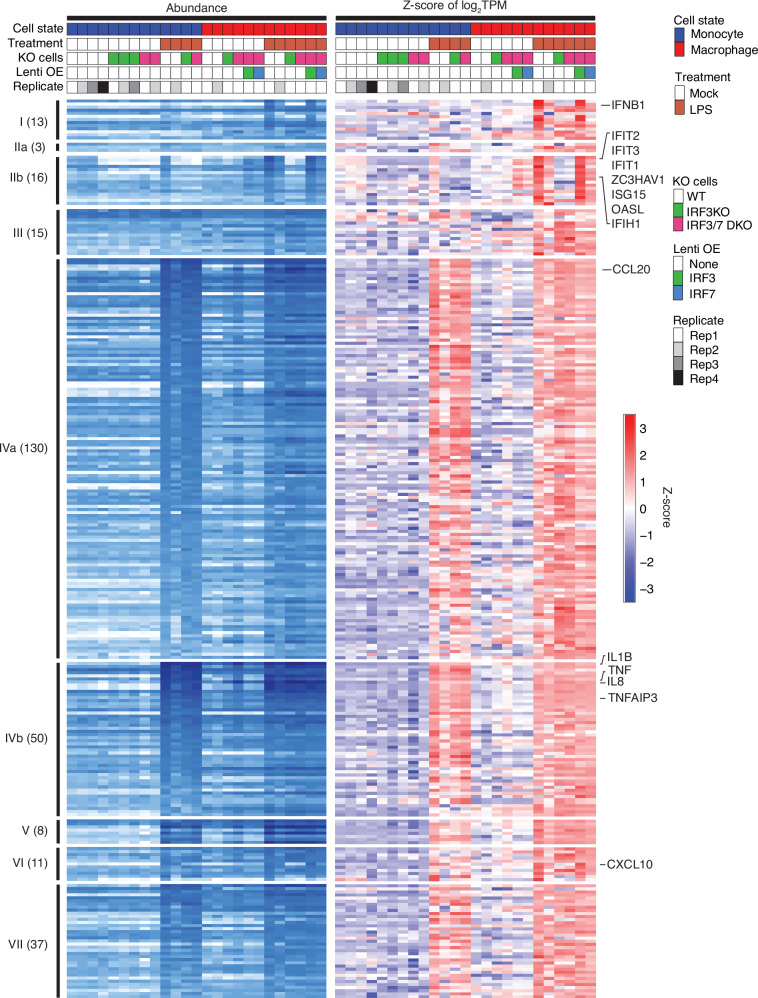
LPS-induced TLR4 activation drives IFNB1 and ISG expression in THP1 macrophages. 283 genes upregulated by LPS in THP1 macrophages showing overlapping induction by various treatments in THP1 monocytes (Fig. 2). Numbers in parentheses denote the total number of overlapping genes identified per group. Several Group I and IIb genes are also induced by LPS in THP1 macrophages, including IFNB1 and multiple ISGs; their induction is IRF3-dependent.

LPS treatment in macrophage THP1 cells yielded 369 upregulated genes (Fig. 1c), 283 of which were also captured by Group I to VII in monocyte THP1 cells (Fig. 7). These included 181 genes of the NF-κB response Group IV, and 29 of the cGAMP-activated Group I and IIb genes, such as *IFNB1*, *IFIT1*, *IFIT2*, and *IFIT3*, indicating an IRF3- and/or IRF7-mediated response along with the dominant LPS-driven NF-κB-mediated response. Co-induction of fast response Group IIb ISGs was weak, mirroring the 18-fold reduced *IFNB1* abundance in TPM compared to cGAMP-treated monocytes. The IRF3 KO macrophage THP1 cells were blunted for *IFNB1* expression and early ISG response.

## Discussion

Dissecting inflammatory gene expression has important implications for drug developers selectively targeting these pathways and thereby controlling specific gene sets for therapeutic intervention. Inhibiting innate-immune-signaling including interferon and cytokine expression will control different gene sets (Group I and IIRGs) compared to blocking interferon- or cytokine-receptor-signaling (Group IIa and IIb). Our approach unmasked early and direct innate immune transcriptional responses from subsequent overlapping autocrine and paracrine receptor-mediated responses.

Group I and Group II genes were induced 2 h post cGAMP or IFN protein stimulation, respectively, sharing a set of 80 genes induced by either treatment (Group IIb). Group I genes included the well-established type I and III IFN genes^62^ requiring IRF3 or IRF7. By treating IFNAR2 KO cells with cGAMP, we identified 29 dual-responsive IIRGs within Group IIb, the remainder representing rapidly induced ISGs, including those with established feed-forward functions (*IRF1*, *IRF7*, *IRF9*, *STAT1*, and *STAT2*). The IIRGs include *IFIT1*, *IFIT2*, *IFIT3*, *ISG15*, *OAS2*, and *IFI44*, which prevent viral replication of infected cells^63^. While these genes were originally described as ISGs, their unexpected induction was previously noted upon expression of mutant and constitutively active IRF3 in human Jurkat T lymphocyte cells unable to produce IFNs^38^. IRF3-dependent but IFN-independent induction of *IFIT1*, *IFIT2*, *IFIT3*, and *ISG15* was also demonstrated in human foreskin fibroblast cells engineered to express STAT1-degrading parainfluenza virus 5 V protein during human cytomegalovirus infection^64^. Importantly, we observed up to 10-fold stronger upregulation of these antiviral genes by cGAMP-mediated IRF3 or IRF7 innate immune activation than by IFNB1 protein exposure. While we used optimized conditions for recording innate immune responses, we note that Vesicular Stomatitis Virus (VSV) infection of THP1 cells yielded similar innate immune response (Group I and II) with respect to transcript abundances after 24 h, including IFNA gene induction^9^.

This study also identified several other IRF-dependent Group I genes, which merit further functional interrogation. OTUD1 was recently characterized as a negative regulator of type I IFN induction by deubiquitinating IRF3^65^. The E3 ubiquitin ligases RNF149^66^ and PELI1^67^ may play similar roles in innate immune regulation. Other Group I genes are implicated in various non-immunomodulatory functions such as neural development (SEMA3D)^68^, collagen assembly (LEPREL1)^69^, and mammalian auditory system development (SIX1)^70^.

KO of IRF1, IRF2, or IRF5 showed no impairment of cGAMP- or LPS-mediated IFN responses in THP1 monocyte or macrophage cells. Perhaps these IRFs require additional cofactors absent in THP1 cells or they play roles in other innate sensing pathways we did not study. IRF5 has been implicated in TLR7-activated IFN induction of THP1 cells^29^, however, we found *TLR7* not expressed (≤2 TPM). We were also unable to note a role of IRF8 in enhancing IFNB1 induction described for human blood monocytes^30^ or impairing STING1 activation noted in THP1 cells^71^. Interestingly, untreated IRF8 KO cells showed highly elevated *CTSG* expression, a serine protease otherwise localized in azurophil granules. The CTSG protease abundance in IRF8 KO cells was so profound that it required additional protease inhibitor treatment to prevent protein degradation during cell lysate preparation. *CTSG* is also highly expressed in mast cells^72^ and proliferating neutrophil progenitors^73^, where *IRF8* expression is absent or modest, respectively. In contrast, plasmacytoid and myeloid dendritic cells^74^ show high *IRF8* expression but do not express *CTSG*. Finally, we found that *IFNB1* induction in THP1 macrophage cells following LPS-mediated activation of TLR4 pathway is dependent on IRF3 but not on IRF1^75^.

A recent study reported that the NF-κB pathway is co-activated by engagement of the CGAS-STING1 in THP1 cells^76^, based on RELA phosphorylation following 2 h treatment with a phosphorothioate-modified cyclic dinucleotide. Using unmodified cGAMP, we and others did not make this observation^77^.

IRF3 or IRF7 KO in human epithelial HT1080 cell line enhanced NF-κB-dependent pro-inflammatory gene induction following LPS treatment^78,79^. Using THP1 monocyte or macrophage cells, our transcriptional responses upon LPS treatment were unaffected by IRF KO or DKO. These discrepancies may be attributed to differences in cellular systems.

IRF9-independent ISGs identified in this study may reflect IFNAR-mediated activation of cryptic DNA binding sites or alternative transcription factor complexes, such as phosphorylated STAT1 homodimers known to bind to GAS elements in the promoter region leading to induction of a subset of ISGs (*ICAM1* and *IRF1*)^12,13,80^. Although primarily induced by IFNγ (*IFNG*), STAT1 homodimers have also been reported to be activated by type I IFNs. We noticed that most of our IRF9-independent ISGs were not induced in HeLa cells following IFNB1 protein treatment. This discrepancy may be attributed to cell-type-specific epigenetic differences and/or alternative transcription and enhancer complexes, or differences in observation time (2 h versus 16 h).

On a cautionary note, while CXCL10 is an established marker of the M1 pro-inflammatory state of macrophages^81^, it is also widely employed as a surrogate marker of the activation of innate immune sensing pathways and IFN protein signaling^82–84^. Activation of a CXCL10 promoter-luciferase reporter construct by NF-κB or IRF3 during Hepatitis C virus infection had been reported previously^85^. We observed induction of *CXCL10* by either LPS, cGAMP, or IFNB1 protein 2 h post treatment, however, the abundance or TPM of *CXCL10* mRNA 2 h post treatments was about 100-fold less compared to 16 or 24 h exposure of THP1 monocytes to cGAMP^8^, VSV^9^ or a 6 h treatment with IFNγ-LPS of PMA-differentiated THP1 macrophages^86^. High level *CXCL10* expression may implicate a form of feed-forward regulation established upon prolonged cGAMP treatment or viral infection partially engaging signaling factors otherwise fully activated upon IFNγ-LPS treatment driving the M0- to M1-transition of macrophages. *CXCL10* regulation appears distinct from the IRF7-driven feed-forward loop considering its modest expression during otherwise high-level *IFNA* and *IFNB1* expression in cGAMP-stimulated IRF7-OE THP1 monocytes.

The highly upregulated genes in this cell culture study correlated well with those induced in circulating leucocytes of viremic patients^87^, though it missed induction of some cell-lineage-specific genes expressed by granulocytes/neutrophils (e.g. *CXCR2*, *FCGR3B*). Therefore, it might be beneficial expanding our approach to other immune cells and cell types in uncovering cell-specific innate immune transcriptional response.

In conclusion, innate immune stimulation followed by transcriptomic analysis using wild-type and isogenic gene KO cell lines identified distinct transcriptional response groups of genes characteristic of the underlying specific signaling pathways engaged upon viral, bacterial, or sterile (i.e. self) PAMP-triggered inflammation. Outlining these pathways and their unique as well as dual- or multiple-regulated gene groups will benefit inflammatory drug discovery. We have summarized these observations in Supplementary Data 5 for use in gene ontology and illustrated its application quantifying which innate immune pathways are active during viral replication in THP1 cells (Supplementary Fig. 11). Given that degenerative or fibrotic medical conditions have a chronic inflammatory component^88^, inhibition of the innate immune response may be broadly beneficial to control disease progression.

## Materials and methods

### Antibodies and reagents

Proteins were detected by Western blotting using antibodies from Cell Signaling Technology, IRF1 8478S, IRF7 4920S, IRF8 5628S, IRF9 76684S, STAT1 9175S, pSTAT1-Y701 9167S, pIRF3-S386 37829S, pIRF3-S396 4947S, pTBK1-S172 5483S, pSTING-S366 19781S, NF-κB-RELA 8242S, NF-κB-pRELA-S536 3033S; R&D Systems, IRF2 AF4049; Abcam, IRF3 ab68481, IRF5 ab33478; Proteintech, GAPDH 60004-1-lg; Sigma-Aldrich, FLAG F3165; Dako, polyclonal goat anti-rabbit Immunoglobulins/HRP P0448, polyclonal goat anti-mouse Immunoglobulins/HRP P0447. Reagents were from InvivoGen, cGAMP vac-nacga23; PBL assay science, recombinant IFNB1 11415; Sigma-Aldrich, LPS L3129-10MG.

### Cell culture

THP1 cells from ATCC TIB-202 were cultured in Gibco RPMI 1640 supplemented with Sigma-Aldrich 10% v/v heat-inactivated fetal bovine serum (FBS), 100 U ml^−1^ penicillin, 100 µg ml^−1^ streptomycin, and 2 mM L-glutamine. HEK293T/17 cells were cultured in Thermo Fisher Scientific high glucose DMEM 11965118 supplemented with 10% v/v FBS, 100 U ml^−1^ penicillin, 100 µg ml^−1^ streptomycin and 2 mM L-glutamine.

### Cell lysis and western blot analysis

Cells were collected by centrifugation and washed with PBS to remove residual culture media. Cell pellets were incubated on ice for 15 min with 50 µl of lysis buffer per million cells. The lysis buffer contained 50 mM Tris-HCl pH 7.5, 150 mM NaCl, 2 mM EDTA, 2% NP40, 0.5 mM DTT, and was supplemented with 1 tablet of cOmplete EDTA-free protease inhibitor cocktail (Roche, 11836170001) and 1 tablet of PhosSTOP (Sigma Aldrich, 4906845001) per 50 ml. Lysis buffer for IRF8 KO cells was further supplemented with 2 mM PMSF and 4 mM AEBSF. Cell lysates were cleared by centrifugation at 13,000 × *g* at 4 °C for 15 min. Supernatant protein concentration was measured using the Thermo Fisher Scientific BCA protein assay kit 23225. For Western blot analysis, 20–40 µg of total protein per sample was diluted in 1× SDS loading dye (1.7% SDS, 5% glycerol, 0.002% bromophenol blue, 60 mM Tris-HCl pH 6.8, 100 mM DTT), separated on a 12% SDS-polyacrylamide gel, and transferred to a nitrocellulose membrane by electroblotting using a Bio-Rad semi-dryer blotter. Membranes were blocked with 5% BSA in 1× TBS-0.1% Tween for 3 h and incubated with the primary antibody overnight at 4 °C followed by three washes in 1× TBS-0.1% Tween. Secondary antibody incubation was performed using horseradish peroxidase-conjugated secondary antibody at room temperature for 3 h followed by three washes of 1× TBS-0.1% Tween. Finally, chemiluminescent detection was performed using a GE Amersham Imager 600 to visualize the blots.

### Generation of THP1 KO cell lines

CRISPR-Cas9-mediated genome editing of THP1 cells was performed as described^89^ with few modifications. Briefly, 2.5 million THP1 cells were pelleted, washed once with PBS at ambient temperature and fresh pre-warmed culture media, and resuspended in 250 µl Gibco Opti-MEM 31985070. 2.5 µg pL-U6-gRNA-GFP and 2.5 µg pRZ-CMV-Cas9 plasmid were combined in a BIO-RAD 0.4 cm-gap sterile electroporation cuvette 1652081 together with the resuspended THP1 cells. Electroporation was performed using BIO-RAD Gene Pulser II using an exponential pulse at 250 V and 950 µF. Electroporated cells were gently resuspended and transferred into a 6-well-plate containing 3.75 ml of pre-warmed 50% THP1-culture-conditioned media supplemented with 20 µM human cGAS inhibitor InvivoGen G140 inh-g140. The inclusion of the cGAS inhibitor improved cell viability during and after transfection. After 24 h, live GFP^+^/mCherry^+^ cells were sorted using a BD FACS Aria device. Double-positive cells were sorted and individually placed into a 96-well-plate containing 70% conditioned media. Colonies were grown until visible to the naked eye, after which they were transferred into larger plates for expansion and subsequently tested for gene KO.

### Lentivirus production and lentiviral gene transduction

Four million HEK293T/17 cells were plated into a 10-cm cell culture plate. Lentiviral particle production was initiated 24 h after seeding by co-transfecting 5 µg Origene pLenti-EF1a-C-Myc-DDK-P2A-Puro empty plasmid PS100120 or pLenti-EF1a-C-Myc-DDK-P2A-Puro-GOI, along with 3.75 µg Addgene psPAX2 12260 and 1.25 µg pMD2.G 12259 packaging plasmids, using Thermo Fisher Scientific Lipofectamine 2000 11668019. The medium was replaced 24 h post-transfection. Viral-particle-containing supernatant was collected 48 and 72 h post-transfection, cleared by centrifugation at 500 × *g* for 10 min at room temperature to remove cellular debris, and then passed through a Fisher Scientific 45 µm filter 09-720-005. The filtrate containing the viral particles was complemented with 1/4 volume of ice-cold System Biosciences PEG-it LV810A and incubated for 24 h at 4 °C. The viral particles were pelleted by centrifugation at 1500 × *g* for 30 min at 4 °C. The supernatant was aspirated, and the viral pellet resuspended in 500 µl of PBS.

Lentiviral transduction was performed by spinfection in a 2 ml final volume in 12-well cell culture plates containing 1 million THP1 cells, 40 µl of virus, and 10 µg ml^−1^ polybrene at 1258 × *g* for 90 min at 37 °C. 24 h after infection, cells were transferred to a 6-well plate containing 2 ml of fresh culture media and incubated for an additional 24 h. Cells were pelleted, resuspended in 3 ml fresh media, and selected with 2 µg ml^−1^ puromycin. Expression of FLAG-tagged proteins was confirmed by Western blot using gene- or FLAG-specific antibodies.

### Plasmids

gRNA plasmids for CRISPR KOs were built following established protocols^89,90^. The gRNA sequence targeting the coding region of the gene of interest was designed using online tool (http://crispor.tefor.net)^91^. The gRNA, with the PAM sequence flanked by constant sequences (5’GGAAAGGACGAAACACCG-gRNA-PAM-TTTAGAGCTAGAAATAGC-3’), was inserted into pL-U6-gRNA-GFP using the ligation-independent assembly method^90^.

For generation of lentiviral overexpression plasmids, the corresponding coding sequence was amplified from cDNA prepared from 20 h IFNB1-protein-treated THP1 cells, introducing AscI/MluI restriction sites for GFP, IRF3, and IRF9, or AscI/NotI restriction sites for IRF7. The GFP PCR product was amplified from Addgene pcDNA5_FRT-GFP 127108. The resulting PCR product and Origene pLenti-EF1a-C-Myc-DDK-P2A-Puro PS100120 were digested with the respective restriction enzymes. Digested plasmids were gel purified and ligated into pLenti-EF1a-C-Myc-DDK-P2A-Puro, followed by sequence verification. All plasmids created or used in this study are available from Addgene.

### RNA isolation

Total RNA for RT-qPCR and RNA-seq analysis was isolated from each sample using TRIzol (Thermo Fisher) following manufacturer’s protocol. Briefly, cells harvested after various treatments were pelleted, washed with PBS, and RNA was extracted using TRIzol (Thermo Fisher).

### RT-qPCR gene expression analysis

RNA isolated from THP1 cells was quantified using RT-qPCR. Briefly, 800 ng RNA was reverse-transcribed into cDNA in a 20 µl final reaction volume using 2.5 µM oligo(dT)_20_ primer and 10 U µl^−1^ Superscript III (Thermo Fisher) for 50 min at 50 °C. Quantitative PCR was performed on an Mx300P qPCR System (Agilent Technologies) using 1/80th of the reverse transcribed material as input for qPCR. Expression of mRNAs was measured in triplicate per sample. Threshold cycle (C_T_) values for selected mRNAs were normalized to TUBA1B C_T_ values and used to calculate ΔC_T_. Relative mRNA expression levels were calculated using the ΔΔC_T_ method (2^ΔΔC^_T_) and shown relative to digitonin only control (Supplementary Figs. 1D, 7A, B) or media (Supplementary Fig. 1E, F, H). Primer sequences are provided in Supplementary Table 1.

### Sequencing and analysis

Oligo(dT)-selected RNA was converted into cDNA using the Illumina Truseq RNA Sample Preparation Kit v2. Sequencing was performed at The Rockefeller University Genomics Resource Center on the Illumina NextSeq 500 platform with 2 × 75 nt paired-end.

Annotation was performed using an in-house custom pipeline. Sequencing file quality was assessed with FastQC software (http://www.bioinformatics.babraham.ac.uk/projects/fastqc). Quality-filtered reads were mapped using BOWTIE^92^ allowing a maximum of two mismatches, against an in-house curated transcript database of the human transcriptome, genome (hg.38). A hierarchical approach was used to assign each read in the order of mRNA, lincRNA, tRNA, miRNA, rRNA, and uncharacterized genomic sequences. Read counts for the mRNA and lincRNA categories were selected for downstream differential gene expression analysis using DESeq2 or normalized to TPM. Data representation and visualization were performed using the R packages ggplot2 and pheatmap.

### Pairwise expression analysis

Pairwise analysis was performed between the following samples: i) Mock-treated KO cell versus mock parental cells in both monocytes and PMA-differentiated cells, and ii) treated versus mock conditions for parental, KO or lentiviral GFP-, IRF3-, or IRF7-overexpressing cells within each treatment group. A pseudo count of 20 was uniformly applied for fold change (FC) calculations. A union of regulated genes (|FC|> 8 for monocyte-to-macrophage comparisons and |FC|> 4 for all others) was generated in each group and combined together for all comparisons. The final gene list for unsupervised clustering analysis included genes with TPM > 300 in at least one sample, resulting in 360 highly regulated genes. The selected 360 genes used for unsupervised clustering analysis are listed in Supplementary Data 3.

### Assessment of IRF dependence in gene groups

Averaged z-scores were used to assess the contribution of individual IRFs to the induction of each gene group. For each sample, z-scores were averaged across seven gene groups (Fig. 2) and subjected to unsupervised clustering to determine dependence of IRF within each group. Most KO samples exhibited averaged z-scores comparable to wild-type sample under the same treatment conditions (Supplementary Fig. 6A).

ISGs impacted by the absence of IRF9 were identified calculating the ratio of IRF9 KO to wild-type for IFNB1-protein-treated samples. Those with a ratio >0.7 representing retention of 70% or greater in induction were classified as IRF9-independent.

Dependence of IRF3 or IRF7 on group I genes (Fig. 3) was identified through pairwise FC analysis comparing cGAMP-treated IRF OE cells to digitonin-treated parental cells. The threshold selected for the classification of group I genes uniquely or commonly upregulated by IRF3 or IRF7 are indicated in Fig. 3 legend. A uniform pseudo counts of 1 was applied across all samples. The corresponding log_2_FC values are also presented in Supplementary Data 3.

### Statistics and reproducibility

RNA-seq replicates were not generated for all the samples in this study due to the extensive scale of the study encompassing numerous KO cell lines and treatment conditions. Samples with available replicates are indicated in Fig. 1B. Samples showing concordant RNA profiles of isogenic lines that were indistinguishable from co-clustered subgroup samples were considered as replicates for DGE analysis. All RT-qPCR data are expressed as mean of two or three technical replicates.

### Reporting summary

Further information on research design is available in the Nature Portfolio Reporting Summary linked to this article.

## Supplementary information

Transparent Peer Review file

Supplementary Information

Reporting Summary

Supplementary Data 1

Supplementary Data 2

Supplementary Data 3

Supplementary Data 4

Supplementary Data 5

Supplementary Data 6

## Data Availability

RNA-seq data are deposited at NCBI Short-Read Archive (SRA) under the BioProject number PRJNA1244336. The following public RNA-seq data series were also used in this study: GSE133139, GSE154596, GSE79272, GSE69906, GSE114966, GSE157052, GSE199674, GSE128113, GSE176261 and GSE130011. Expression values for all samples generated, analyzed, and included in this study (Supplementary Data 1-5), and numerical source data for the RT-qPCR plots (Supplementary Data 6), are publicly available in the Dryad repository [10.5061/dryad.v15dv428j]. Uncropped blot images are provided in Supplementary Information as Supplementary Figs. 12–17.

## Code availability

No custom computer codes were generated in this study.

## References

[1] Negishi, H., Taniguchi, T. & Yanai, H. The Interferon (IFN) class of cytokines and the IFN Regulatory Factor (IRF) transcription factor family. *Cold Spring Harb. Perspect. Biol.* **10**, a028423 (2018). 28963109 10.1101/cshperspect.a028423 PMC6211389

[2] Mogensen, T. H. Pathogen recognition and inflammatory signaling in innate immune defenses. *Clin. Microbiol. Rev.* **22**, 240–273 (2009). 19366914 10.1128/CMR.00046-08 PMC2668232

[3] Pandolfi, F., Altamura, S., Frosali, S. & Conti, P. Key role of DAMP in inflammation, cancer, and tissue repair. *Clin. Ther.* **38**, 1017–1028 (2016). 27021609 10.1016/j.clinthera.2016.02.028

[4] Fitzgerald, K. A. & Kagan, J. C. Toll-like receptors and the control of immunity. *Cell* **180**, 1044–1066 (2020). 32164908 10.1016/j.cell.2020.02.041 PMC9358771

[5] Rehwinkel, J. & Gack, M. U. RIG-I-like receptors: their regulation and roles in RNA sensing. *Nat. Rev. Immunol.* **20**, 537–551 (2020). 32203325 10.1038/s41577-020-0288-3 PMC7094958

[6] Hopfner, K.-P. & Hornung, V. Molecular mechanisms and cellular functions of cGAS–STING signalling. *Nat. Rev. Mol. Cell Biol.* **21**, 501–521 (2020). 32424334 10.1038/s41580-020-0244-x

[7] Kusiak, A. & Brady, G. Bifurcation of signalling in human innate immune pathways to NF-kB and IRF family activation. *Biochem. Pharmacol.* **205**, 115246 (2022). 36088989 10.1016/j.bcp.2022.115246

[8] Rothamel, K. et al. ELAVL1 primarily couples mRNA stability with the 3′ UTRs of interferon-stimulated genes. *Cell Rep.* **35**, 109178–109178 (2021). 34038724 10.1016/j.celrep.2021.109178 PMC8225249

[9] Cao, L. et al. SAFA facilitates chromatin opening of immune genes through interacting with anti-viral host RNAs. *PLOS Pathog.* **18**, e1010599 (2022). 35658050 10.1371/journal.ppat.1010599 PMC9200321

[10] Sokol, C. L. & Luster, A. D. The chemokine system in innate immunity. *Cold Spring Harb. Perspect. Biol.* **7**, a016303 (2015). 25635046 10.1101/cshperspect.a016303 PMC4448619

[11] Lazear, H. M., Schoggins, J. W. & Diamond, M. S. Shared and distinct functions of type I and type III interferons. *Immunity* **50**, 907–923 (2019). 30995506 10.1016/j.immuni.2019.03.025 PMC6839410

[12] Ivashkiv, L. B. & Donlin, L. T. Regulation of type I interferon responses. *Nat. Rev. Immunol.* **14**, 36–49 (2014). 24362405 10.1038/nri3581 PMC4084561

[13] Levy, D. E., Kessler, D. S., Pine, R. & Darnell, J. E. Cytoplasmic activation of ISGF3, the positive regulator of interferon-alpha-stimulated transcription, reconstituted in vitro. *Genes Dev.* **3**, 1362–1371 (1989). 2606351 10.1101/gad.3.9.1362

[14] Ohkuri, T. et al. Intratumoral administration of cGAMP transiently accumulates potent macrophages for anti-tumor immunity at a mouse tumor site. *Cancer Immunol., Immunother.* **66**, 705–716 (2017). 28243692 10.1007/s00262-017-1975-1 PMC11028681

[15] Sharif, O., Bolshakov, V. N., Raines, S., Newham, P. & Perkins, N. D. Transcriptional profiling of the LPS induced NF-κB response in macrophages. *BMC Immunol.* **8**, 1–1 (2007). 17222336 10.1186/1471-2172-8-1 PMC1781469

[16] Zheng, Z., Jia, S., Shao, C. & Shi, Y. Irradiation induces cancer lung metastasis through activation of the cGAS–STING–CCL5 pathway in mesenchymal stromal cells. *Cell Death Dis.* **11**, 326 (2020). 32382015 10.1038/s41419-020-2546-5 PMC7206094

[17] Hughes, C. E. & Nibbs, R. J. B. A guide to chemokines and their receptors. *Febs J.* **285**, 2944–2971 (2018). 29637711 10.1111/febs.14466 PMC6120486

[18] Liu, T., Zhang, L., Joo, D. & Sun, S.-C. NF-κB signaling in inflammation. *Sig. Transduct. Target. Ther.* **2**, 17023 (2017). 10.1038/sigtrans.2017.23 PMC5661633 29158945

[19] Chan, A. H. & Schroder, K. Inflammasome signaling and regulation of interleukin-1 family cytokines. *J. Exp. Med.* **217**, e20190314 (2019). 10.1084/jem.20190314 PMC7037238 31611248

[20] Antonczyk, A. et al. Direct inhibition of IRF-dependent transcriptional regulatory mechanisms associated with disease. *Front. Immunol.* **10**, 1176 (2019). 31178872 10.3389/fimmu.2019.01176 PMC6543449

[21] Fujita, T. et al. Evidence for a nuclear factor(s), IRF-1, mediating induction and silencing properties to human IFN-beta gene regulatory elements. *EMBO J.* **7**, 3397–3405 (1988). 2850164 10.1002/j.1460-2075.1988.tb03213.x PMC454838

[22] Reis, L. F., Ruffner, H., Stark, G., Aguet, M. & Weissmann, C. Mice devoid of interferon regulatory factor 1 (IRF-1) show normal expression of type I interferon genes. *EMBO J.* **13**, 4798–4806 (1994). 7957048 10.1002/j.1460-2075.1994.tb06805.x PMC395418

[23] Honda, K. et al. IRF-7 is the master regulator of type-I interferon-dependent immune responses. *Nature* **434**, 772–777 (2005). 15800576 10.1038/nature03464

[24] Gao, M., Qi, Y. & Zhang, J. IRF1 amplifies HSV-1-triggered antiviral innate immunity in a feed-forward manner. *Cell insight* **4**, 100255 (2025). 40586041 10.1016/j.cellin.2025.100255 PMC12205802

[25] Marié, I., Durbin, J. E. & Levy, D. E. Differential viral induction of distinct interferon-α genes by positive feedback through interferon regulatory factor-7. *EMBO J.* **17**, 6660–6669 (1998). 9822609 10.1093/emboj/17.22.6660 PMC1171011

[26] Sato, M. et al. Positive feedback regulation of type I IFN genes by the IFN-inducible transcription factor IRF-7. *FEBS Lett.* **441**, 106–110 (1998). 9877175 10.1016/s0014-5793(98)01514-2

[27] Ito, T., Kanzler, H., Duramad, O., Cao, W. & Liu, Y.-J. Specialization, kinetics, and repertoire of type 1 interferon responses by human plasmacytoid predendritic cells. *Blood* **107**, 2423–2431 (2006). 16293610 10.1182/blood-2005-07-2709

[28] Heinz, L. X. et al. TASL is the SLC15A4-associated adaptor for IRF5 activation by TLR7–9. *Nature* **581**, 316–322 (2020). 32433612 10.1038/s41586-020-2282-0 PMC7610944

[29] Schoenemeyer, A. et al. The interferon regulatory factor, IRF5, is a central mediator of toll-like receptor 7 signaling*. *J. Biol. Chem.* **280**, 17005–17012 (2005). 15695821 10.1074/jbc.M412584200

[30] Li, P. et al. IRF8 and IRF3 cooperatively regulate rapid interferon-β induction in human blood monocytes. *Blood* **117**, 2847–2854 (2011). 21228327 10.1182/blood-2010-07-294272

[31] Latz, E. et al. The LPS receptor generates inflammatory signals from the cell surface. *J. Endotoxin Res.* **9**, 375–380 (2003). 14733724 10.1179/096805103225003303

[32] Kagan, J. C. & Medzhitov, R. Phosphoinositide-mediated adaptor recruitment controls toll-like receptor signaling. *Cell* **125**, 943–955 (2006). 16751103 10.1016/j.cell.2006.03.047

[33] Zanoni, I. et al. CD14 Controls the LPS-Induced Endocytosis of Toll-like Receptor 4. *Cell* **147**, 868–880 (2011). 22078883 10.1016/j.cell.2011.09.051 PMC3217211

[34] Kagan, J. C. et al. TRAM couples endocytosis of Toll-like receptor 4 to the induction of interferon-β. *Nat. Immunol.* **9**, 361–368 (2008). 18297073 10.1038/ni1569 PMC4112825

[35] Akira, S. & Takeda, K. Toll-like receptor signalling. *Nat. Rev. Immunol.* **4**, 499–511 (2004). 15229469 10.1038/nri1391

[36] Wang, L. et al. The multiple roles of interferon regulatory factor family in health and disease. *Sig. Transduct. Target. Ther.* **9**, 282 (2024). 10.1038/s41392-024-01980-4 PMC11486635 39384770

[37] Swaraj, S. & Tripathi, S. Interference without interferon: interferon-independent induction of interferon-stimulated genes and its role in cellular innate immunity. *mBio* **15**, e02582-24 (2024). 39302126 10.1128/mbio.02582-24 PMC11481898

[38] Grandvaux, N. et al. Transcriptional profiling of interferon regulatory factor 3 target genes: direct involvement in the regulation of interferon-stimulated genes. *J. Virol.* **76**, 5532–5539 (2002). 11991981 10.1128/JVI.76.11.5532-5539.2002 PMC137057

[39] Tsuchiya, S. et al. Establishment and characterization of a human acute monocytic leukemia cell line (THP-1). *Int. J. Cancer* **26**, 171–176 (1980). 6970727 10.1002/ijc.2910260208

[40] Chanput, W., Mes, J. J. & Wichers, H. J. THP-1 cell line: an in vitro cell model for immune modulation approach. *Int. Immunopharmacol.* **23**, 37–45 (2014). 25130606 10.1016/j.intimp.2014.08.002

[41] Mizuno, K., Toyoda, Y., Fukami, T., Nakajima, M. & Yokoi, T. Stimulation of pro-inflammatory responses by mebendazole in human monocytic THP-1 cells through an ERK signaling pathway. *Arch. Toxicol.* **85**, 199–207 (2011). 20848085 10.1007/s00204-010-0584-y

[42] Martinez, O. et al. Ebola virus exploits a monocyte differentiation program to promote its entry. *J. Virol.* **87**, 3801–3814 (2013). 23345511 10.1128/JVI.02695-12 PMC3624207

[43] Zakharova, E., Grandhi, J., Wewers, M. D. & Gavrilin, M. A. Mycoplasma suppression of THP-1 cell TLR responses is corrected with antibiotics. *PLoS ONE* **5**, e9900 (2010). 20360862 10.1371/journal.pone.0009900 PMC2845629

[44] Tóth, R. et al. Investigation of Candida parapsilosis virulence regulatory factors during host-pathogen interaction. *Sci. Rep.* **8**, 1346 (2017). 10.1038/s41598-018-19453-4 PMC5777994 29358719

[45] Nascimento, C. R., Fernandes, N. A. R., Maldonado, L. A. G. & Junior, C. R. Comparison of monocytic cell lines U937 and THP-1 as macrophage models for in vitro studies. *Biochem. Biophys. Rep.* **32**, 101383 (2022). 36420419 10.1016/j.bbrep.2022.101383 PMC9677084

[46] Mitjans, M. et al. Use of IL-8 release and p38 MAPK activation in THP-1 cells to identify allergens and to assess their potency in vitro. *Toxicol. Vitr* **24**, 1803–1809 (2010). 10.1016/j.tiv.2010.06.001 20541004

[47] Chanput, W., Mes, J. J., Savelkoul, H. F. J. & Wichers, H. J. Characterization of polarized THP-1 macrophages and polarizing ability of LPS and food compounds. *Food Funct.* **4**, 266–276 (2013). 23135314 10.1039/c2fo30156c

[48] Daigneault, M., Preston, J. A., Marriott, H. M., Whyte, M. K. B. & Dockrell, D. H. The identification of markers of macrophage differentiation in PMA-stimulated THP-1 cells and monocyte-derived macrophages. *PLoS ONE* **5**, e8668 (2010). 20084270 10.1371/journal.pone.0008668 PMC2800192

[49] Park, E. K. et al. Optimized THP-1 differentiation is required for the detection of responses to weak stimuli. *Inflamm. Res.* **56**, 45–50 (2007). 17334670 10.1007/s00011-007-6115-5

[50] Stok, J. E. et al. RNA sensing via the RIG-I-like receptor LGP2 is essential for the induction of a type I IFN response in ADAR1 deficiency. *EMBO J.* **41**, e109760 (2022). 35156720 10.15252/embj.2021109760 PMC8922249

[51] Jønsson, K. L. et al. IFI16 is required for DNA sensing in human macrophages by promoting production and function of cGAMP. *Nat. Commun.* **8**, 14391 (2017). 28186168 10.1038/ncomms14391 PMC5309897

[52] Crowl, J. T. & Stetson, D. B. SUMO2 and SUMO3 redundantly prevent a noncanonical type I interferon response. *Proc. Natl. Acad. Sci.* **115**, 6798–6803 (2018). United States Am. 29891701 10.1073/pnas.1802114115 PMC6042150

[53] Zhang, L. et al. Human IFNAR2 mutant generated by CRISPR/Cas9-induced exon skipping upregulates a subset of tonic-like interferon-stimulated genes upon IFNβ stimulation. *J. Interf. Cytokine Res.* **42**, 580–589 (2022). 10.1089/jir.2022.0158 36346319

[54] Platanitis, E. et al. A molecular switch from STAT2-IRF9 to ISGF3 underlies interferon-induced gene transcription. *Nat. Commun.* **10**, 2921 (2019). 31266943 10.1038/s41467-019-10970-y PMC6606597

[55] Wüst, S., Schad, P., Burkart, S. & Binder, M. Comparative analysis of six IRF family members in alveolar epithelial cell-intrinsic antiviral responses. *Cells* **10**, 2600 (2021). 34685580 10.3390/cells10102600 PMC8533862

[56] Schoggins, J. W. et al. A diverse range of gene products are effectors of the type I interferon antiviral response. *Nature* **472**, 481–485 (2011). 21478870 10.1038/nature09907 PMC3409588

[57] Kane, M. et al. Identification of interferon-stimulated genes with antiretroviral activity. *Cell Host Microbe* **20**, 392–405 (2016). 27631702 10.1016/j.chom.2016.08.005 PMC5026698

[58] Panne, D., Maniatis, T. & Harrison, S. C. An atomic model of the interferon-β enhanceosome. *Cell* **129**, 1111–1123 (2007). 17574024 10.1016/j.cell.2007.05.019 PMC2020837

[59] Tamura, T., Kurotaki, D. & Koizumi, S. Regulation of myelopoiesis by the transcription factor IRF8. *Int. J. Hematol.* **101**, 342–351 (2015). 25749660 10.1007/s12185-015-1761-9

[60] Kurotaki, D. et al. IRF8 inhibits C/EBPα activity to restrain mononuclear phagocyte progenitors from differentiating into neutrophils. *Nat. Commun.* **5**, 4978 (2014). 25236377 10.1038/ncomms5978

[61] Urin, V., Shemesh, M. & Schreiber, G. CRISPR/Cas9-based knockout strategy elucidates components essential for type 1 interferon signaling in human HeLa cells. *J. Mol. Biol.* **431**, 3324–3338 (2019). 31207241 10.1016/j.jmb.2019.06.007

[62] Österlund, P. I., Pietilä, T. E., Veckman, V., Kotenko, S. V. & Julkunen, I. IFN regulatory factor family members differentially regulate the expression of type III IFN (IFN-λ) genes. *J. Immunol.* **179**, 3434–3442 (2007). 17785777 10.4049/jimmunol.179.6.3434

[63] Schoggins, J. W. Interferon-stimulated genes: what do they all do?. *Annu. Rev. Virol.* **6**, 567–584 (2019). 31283436 10.1146/annurev-virology-092818-015756

[64] Ashley, C. L., Abendroth, A., McSharry, B. P. & Slobedman, B. Interferon-Independent Upregulation of Interferon-Stimulated Genes during Human Cytomegalovirus Infection is Dependent on IRF3 Expression. *Viruses* **11**, 246 (2019). 30871003 10.3390/v11030246 PMC6466086

[65] Zhang, Z., Wang, D., Wang, P., Zhao, Y. & You, F. OTUD1 negatively regulates type I IFN induction by disrupting noncanonical ubiquitination of IRF3. *J. Immunol.* **204**, 1904–1918 (2020). 32075857 10.4049/jimmunol.1900305

[66] Yang, X.-A. et al. RNF149 negatively regulates LPS/TLR4 signal transduction by ubiquitination-mediated CD63 degradation. *Heliyon* **10**, e34350 (2024). 39104473 10.1016/j.heliyon.2024.e34350 PMC11298846

[67] Xiao, Y. et al. Peli1 negatively regulates type I interferon induction and antiviral immunity in the CNS. *Cell Biosci.* **5**, 34 (2015). 26131354 10.1186/s13578-015-0024-z PMC4486122

[68] Liu, Y. et al. Semaphorin3D guides retinal axons along the dorsoventral axis of the tectum. *J. Neurosci.* **24**, 310–318 (2004). 14724229 10.1523/JNEUROSCI.4287-03.2004 PMC6730002

[69] Tiainen, P., Pasanen, A., Sormunen, R. & Myllyharju, J. Characterization of recombinant human prolyl 3-hydroxylase isoenzyme 2, an enzyme modifying the basement membrane collagen IV*. *J. Biol. Chem.* **283**, 19432–19439 (2008). 18487197 10.1074/jbc.M802973200

[70] Zheng, W. et al. The role of Six1 in mammalian auditory system development. *Development* **130**, 3989–4000 (2003). 12874121 10.1242/dev.00628 PMC3873880

[71] Luo, W.-W. et al. Transcription-independent regulation of STING activation and innate immune responses by IRF8 in monocytes. *Nat. Commun.* **13**, 4822 (2022). 35973990 10.1038/s41467-022-32401-1 PMC9381507

[72] Bahri, R. et al. Human melanoma-associated mast cells display a distinct transcriptional signature characterized by an upregulation of the complement component 3 that correlates with poor prognosis. *Front. Immunol.* **13**, 861545 (2022). 35669782 10.3389/fimmu.2022.861545 PMC9163391

[73] Encabo, H. H. et al. Loss of TET2 in human hematopoietic stem cells alters the development and function of neutrophils. *Cell Stem Cell* **30**, 781–799.e9 (2023). 37267914 10.1016/j.stem.2023.05.004 PMC12356769

[74] Choi, J. et al. Haemopedia RNA-seq: a database of gene expression during haematopoiesis in mice and humans. *Nucleic Acids Res.* **47**, D780–D785 (2019). 30395284 10.1093/nar/gky1020 PMC6324085

[75] Song, R. et al. IRF1 governs the differential interferon-stimulated gene responses in human monocytes and macrophages by regulating chromatin accessibility. *Cell Rep.* **34**, 108891–108891 (2021). 33761354 10.1016/j.celrep.2021.108891 PMC8300000

[76] Balka, K. R. et al. TBK1 and IKKε Act Redundantly to Mediate STING-Induced NF-κB Responses in Myeloid Cells. *Cell Rep.* **31**, 107492 (2020). 32268090 10.1016/j.celrep.2020.03.056

[77] Zhang, L. et al. NF-κB activation enhances STING signaling by altering microtubule-mediated STING trafficking. *Cell Rep.* **42**, 112185 (2023). 36857187 10.1016/j.celrep.2023.112185

[78] Popli, S. et al. IRF3 inhibits nuclear translocation of NF-κB to prevent viral inflammation. *Proc. Natl. Acad. Sci.* **119**, e2121385119 (2022). 36067309 10.1073/pnas.2121385119 PMC9478676

[79] Fan, S., Popli, S., Chakravarty, S., Chakravarti, R. & Chattopadhyay, S. Non-transcriptional IRF7 interacts with NF-κB to inhibit viral inflammation. *J. Biol. Chem.* **300**, 107200 (2024). 38508315 10.1016/j.jbc.2024.107200 PMC11040127

[80] Michalska, A., Blaszczyk, K., Wesoly, J. & Bluyssen, H. A. R. A positive feedback amplifier circuit that regulates interferon (IFN)-stimulated gene expression and controls type I and type II IFN responses. *Front. Immunol.* **9**, 1135 (2018). 29892288 10.3389/fimmu.2018.01135 PMC5985295

[81] Genin, M., Clement, F., Fattaccioli, A., Raes, M. & Michiels, C. M1 and M2 macrophages derived from THP-1 cells differentially modulate the response of cancer cells to etoposide. *BMC Cancer* **15**, 577 (2015). 26253167 10.1186/s12885-015-1546-9 PMC4545815

[82] Li, T. et al. Phosphorylation and chromatin tethering prevent cGAS activation during mitosis. *Science* **371**, eabc5386 (2021). 33542149 10.1126/science.abc5386 PMC8171060

[83] Makuch, E., Jasyk, I., Kula, A., Lipiński, T. & Siednienko, J. IFNβ-induced CXCL10 chemokine expression is regulated by Pellino3 ligase in monocytes and macrophages. *Int. J. Mol. Sci.* **23**, 14915 (2022). 36499241 10.3390/ijms232314915 PMC9741470

[84] Lama, L. et al. Development of human cGAS-specific small-molecule inhibitors for repression of dsDNA-triggered interferon expression. *Nat. Commun.* **10**, 2261 (2018). 10.1038/s41467-019-08620-4 PMC6529454 31113940

[85] Brownell, J. et al. Direct, interferon-independent activation of the CXCL10 promoter by NF-κB and interferon regulatory factor 3 during hepatitis C virus infection. *J. Virol.* **88**, 1582–1590 (2014). 24257594 10.1128/JVI.02007-13 PMC3911583

[86] Green, I. D. et al. Macrophage development and activation involve coordinated intron retention in key inflammatory regulators. *Nucleic Acids Res.* **48**, 6513–6529 (2020). 32449925 10.1093/nar/gkaa435 PMC7337907

[87] Steinbrink, J. M. et al. The host transcriptional response to Candidemia is dominated by neutrophil activation and heme biosynthesis and supports novel diagnostic approaches. *Genome Med.* **13**, 108 (2021). 34225776 10.1186/s13073-021-00924-9 PMC8259367

[88] Wick, G. et al. The immunology of fibrosis. *Immunology* **31**, 107–135 (2013). 10.1146/annurev-immunol-032712-095937 23516981

[89] Schmidt, T., Schmid-Burgk, J. L., Ebert, T. S., Gaidt, M. M. & Hornung, V. Designer nuclease-mediated generation of knockout THP1 cells. *Methods Mol. Biol.* **1338**, 261–272 (2015). 10.1007/978-1-4939-2932-0_19 26443227

[90] Schmidt, T., Schmid-Burgk, J. L. & Hornung, V. Synthesis of an arrayed sgRNA library targeting the human genome. *Sci. Rep.* **5**, 14987 (2015). 26446710 10.1038/srep14987 PMC4597219

[91] Concordet, J.-P. & Haeussler, M. CRISPOR: intuitive guide selection for CRISPR/Cas9 genome editing experiments and screens. *Nucleic Acids Res.* **46**, W242–W245 (2018). 29762716 10.1093/nar/gky354 PMC6030908

[92] Langmead, B., Trapnell, C., Pop, M. & Salzberg, S. L. Ultrafast and memory-efficient alignment of short DNA sequences to the human genome. *Genome Biol.* **10**, R25 (2009). 19261174 10.1186/gb-2009-10-3-r25 PMC2690996

