# Supplementary material for: Dissection of innate-immune-ligand- and interferon-protein-mediated transcriptional responses in human THP1 cell states
Source: Commun Biol. 2026 Feb 12;9:239. doi: 10.1038/s42003-025-09343-7 (PMC12901304; doi:10.1038/s42003-025-09343-7)
Supplement: Supplementary file 2 — Supplementary Information [file 42003_2025_9343_MOESM2_ESM.pdf]

Supplementary Information

Dissection of innate-immune-ligand- and interferon-protein-mediated transcriptional responses in human THP1 cell states

Lodoe Lama[1], Pavel Morozov[1], Aitor Garzia[1], Thomas Tuschl[1]

[1]Laboratory for RNA Molecular Biology, The Rockefeller University, 1230 York Ave, Box 186, New York, NY 10065, USA

Correspondence to: ttuschl@rockefeller.edu

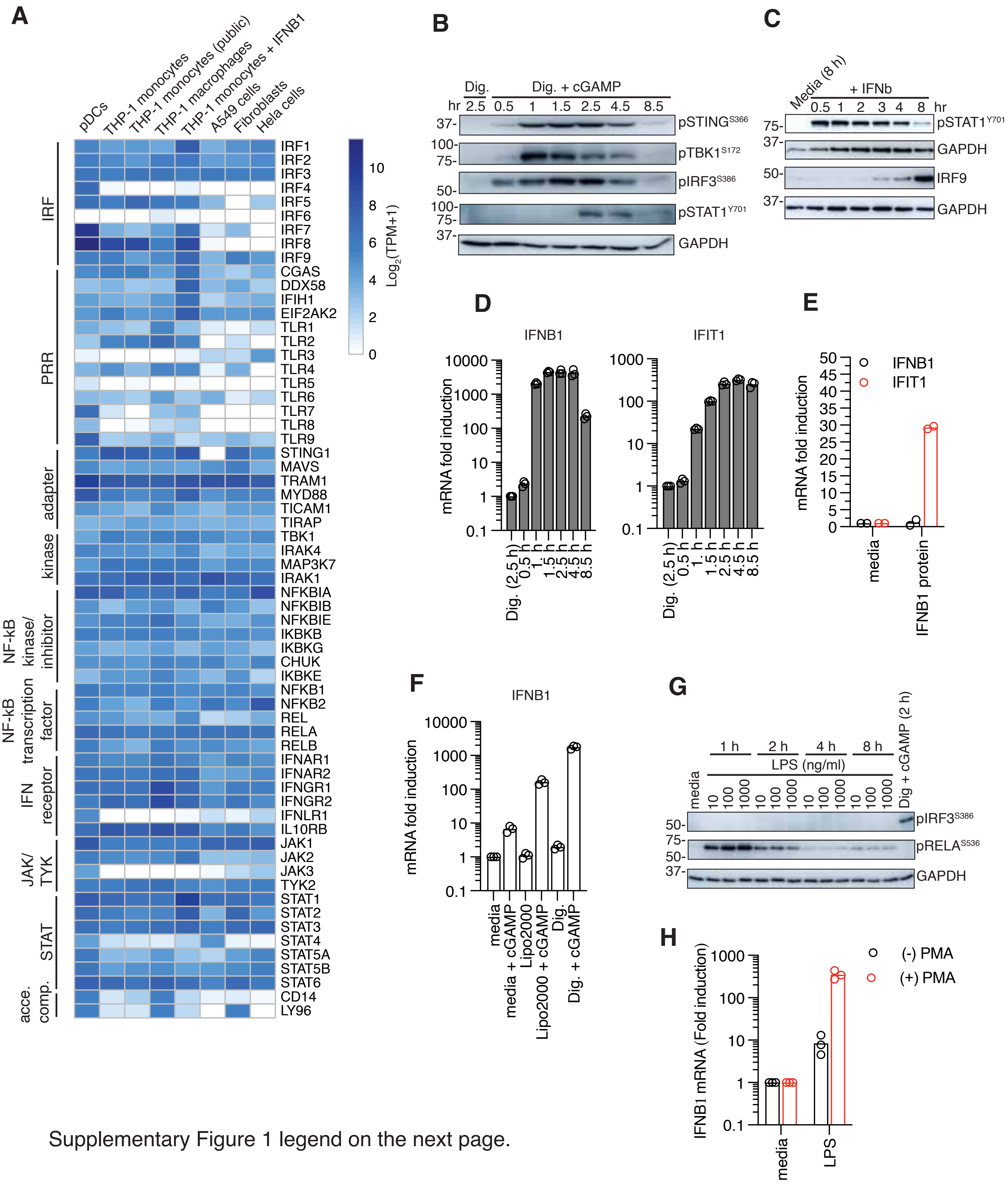

Supplementary Figure 1 legend on the next page.

Supplementary Figure 1: THP1 cells are suitable for studying innate immune sensing pathways. **A.** Comparison of transcript abundance for IRFs, various innate pattern recognition receptors (PRRs), adapters, interferon (IFN) receptors, and other key intermediary signaling and accessory components (acce. comp.) across different cell lines. Plasmacytoid dendritic cells (pDCs) (SRR3229088), THP1 monocytes (public) (GSM3161742), A549 cells (SRR12243954), fibroblast cells (GSM1712278), and HeLa cells (SRR 9336621) were from public datasets. Others were from this study. Genes selected for heatmap visualization are listed in Supplementary Data 1. **B.** Time-course analysis of cGAMP treatment in THP1 monocytes, examining phosphorylation of key signaling intermediates. **C.** Time-course analysis of IFNB1 protein-mediated IFNAR signaling in THP1 monocytes, assessed by western blot (WB) detection of STAT1 phosphorylation and IRF9 protein expression. **D.** Time-course analysis of cGAMP treatment in THP1 monocytes examining terminal gene induction via RT-qPCR analysis of the IFNB1 and IFIT1 mRNA expression. **E.** RT-qPCR analysis of IFNB1 and IFIT1 mRNAs in THP1 monocytes treated with IFNB1 protein for 2 h. **F.** RT-qPCR analysis of IFNB1 mRNAs in THP1 monocytes treated with 10 μg ml^{-1} cGAMP using different delivery methods. **G.** Time-course and dose-dependent analysis of NF-κB pathway activation in THP1 monocytes following LPS stimulation, monitored via RELA phosphorylation. **H.** RT-qPCR analysis of IFNB1 mRNA expression in monocyte or PMA-differentiated macrophage THP1 cells treated with 100 ng ml^{-1} LPS. RT-qPCR data are presented as mean of two or three technical replicates.TPM, transcripts per million; Lipo2000, Lipofectamine 2000; Dig., digitonin.

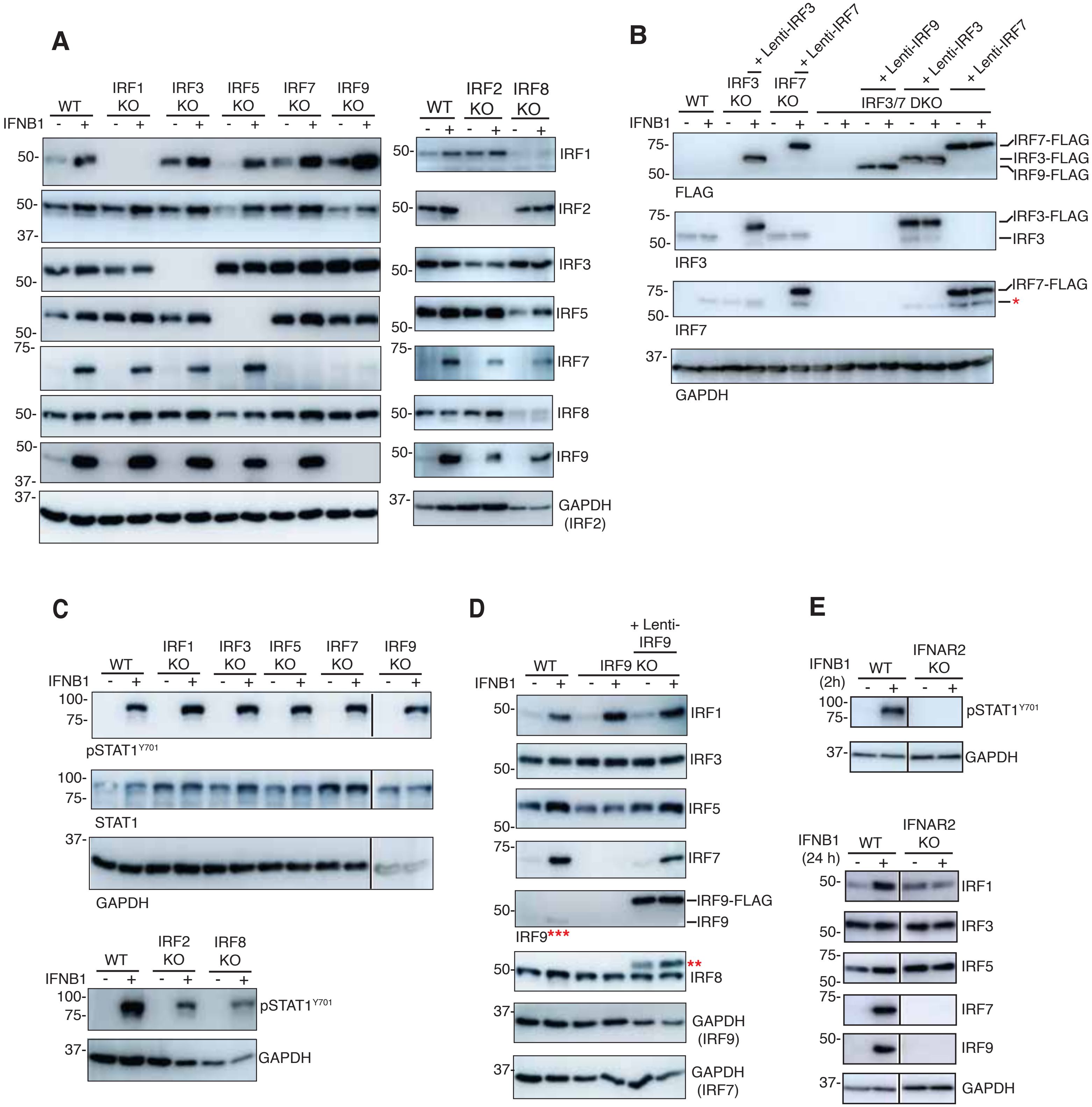

Supplementary Figure 2 legend on the next page.

Supplementary Figure 2: WB analysis and validation of IRF and IFNAR2 KO THP1 monocytes revealed IRF9-dependent and IRF9-independent IFN-inducible IRFs. **A.** Lysates from various KO cells with or without IFNB1 protein treatment (24 h) were analyzed by WB using indicated antibodies. IRF7 induction requires IRF9, while IRF1 induction occurs independently of IRF9. **B.** Lentiviral transduction-mediated reconstitution of IRF3 or IRF7 into single or double KOs of IRF3 and IRF7. Protein re-expression in lentivirus-transduced cells was monitored using the indicated antibodies. * denotes non-specific detection of overexpressed IRF3 and IRF7. **C.** WB analysis of STAT1 phosphorylation in lysates from IFNB1 protein-treated KO cells indicate preserved IFNAR signaling in cells lacking the indicated IRFs. **D.** Lentivirus-mediated reintroduction of IRF9 into IRF9-deficient cells reconfirms IRF9-dependent and IRF9-independent IFN-inducible IRFs. ** indicates non-specific binding of overexpressed IRF9 by IRF8 antibody. *** Only half the lysate was loaded for lanes 5 and 6 in the IRF9 blot to avoid signal saturation due to IRF9 overexpression. **E.** Confirmation of IFNAR2 KO cells by the absence of IFNB1 protein-mediated STAT1 phosphorylation (upper panel) and non-induction of IFN-responsive IRFs (lower panel).

IRF1

WT tctgtgttctctttacagCCAACATGCCCATCACTCGGATGCGCATGAGACCCTGGCTAGAGATGCAGATT
KO (clone A18) TCTGTGTTCTCTTTACAGCCAACATGCCCAT--CTCGGATGCGCATGAGACCCTGGCTAGAGATGCAGATT

2 bp deletion

IRF2

WT ccctcaagGGCACCATGCCGGTGGAAAGGATGCGCATGCGCCCGTGGCTGGAGGAGCAGATAAACTCCAACACGATCCCGGGGCTCAAGT
KO (A5) ccctcaagGGCACCATGCCGGTGGAAAGGATGCGCATGCGCCCGTGGCTGGAGGAGCAGATAAACTCCAACACGATCCCGGGGCTCAAGT

2 bp deletion

WT ccctcaagGGCACCATGCCGGTGGAAAGGATGCGCATGCGC--GTGGCTGGAGGAGCAGATAAACTCCAACACGATCCCGGGGCTCAAGT
KO (A5) ccctcaagGGCACCATGCCGGTGGAAAGGAT-----------------------------------CCAACACGATCCCGGGGCTCAAGT

35 bp deletion

IRF3

WT gctgctgccgctgctcagAGTGGGAGTTCGAGGTGACAGCCTTCTACCGGGGCCGCCAAGTCTTCCAGCAGACCATCTCCTGCCCGGAGGGCCTG
KO (clone 4) GCTGCTGCCGCTGCTCA---GAGGGCCTG

69 bp deletion

WT gctgctgccgctgctcagAGTGGGAGTTCGAGGTGACAGCCTTCT-ACCGGGGCCGCCAAGTCTTCCAGCAGACCATCTCCTGCCCGGAGGGCCTG
KO (clone 4) GCTGCTGCCGCTGCTCAGAGTGGGAGTTCGAGGTGACAGCCTTCTAACCGGGGCCGCCAAGTCTTCCAGCAGACCATCTCCTGCCCGGAGGGCCTG

1 bp insertion

IRF5

WT GCCCTTAACAAGAGCCGGGACTTCCGCCTCATCTACGACGGGCCCCGGGACATGCCACCTCAGCCC
KO (clone LB5) GCCCTTAACAAGAGCCGGGACTTCCGCCTCA--------GGGCCCCGGGACATGCCACCTCAGCCC

8 bp deletion

WT GCCCTTAACAAGAGCCGGGACTTCCGCCTCATCTA-CGACGGGCCCCGGGACATGCCACCTCAGCCC
KO (clone LB5) GCCCTTAACAAGAGCCGGGACTTCCGCCTCATCTATCGACGGGCCCCGGGACATGCCACCTCAGCCC

1 bp insertion

IRF7

WT TGCGCAGCACGCGTCGCTTCGTGATGCTGCGGGATAACTCGGGGGACCCGGCCGACCCGCACAAGGTGTACGCGCTCA
KO (clone B50) TGCG--ACCCGCACAAGGTGTACGCGCTCA

50 bp deletion

WT gttctgtccccctcttcagGCCTGGGCTGTGGCCCGCGGCAGGTGGCCGCCTAGCAGCAGGGGAGGTGGCCCGCCCCCCGAGGCTGAGACTGCG
GAGCGCGCCGGCTGGAAAACCAACTTCCGCTGCGCACTGCGCAGCACGCGTCGCTTCGTGATGCTGCGGGATAACTCGGGGGACCCGGCCGACC
CGCACAAGGTGTACGCGCTCAGCCGGGAGCTGTGCTGGCGAGgtgagcgacgccggctgccaagggcgttggcattcgtcctgcttgggcaaag
gggaacattctctgggtcagatttgtggttttagaagctgttctgggagaggctgccagcaggcccaggacaggtggagagccacatccccacc

KO (clone B50) gttctgtccccctcttcagGC---------------------//---cacatccccacc

343 bp deletion

IRF8

WT tctgtctttccaagGATGTGTGACCGGAATGGTGGT-CGGCGGCTTCGACAGTGGCTGATCGAGCAGATTGA
KO (clone 2) TCTGTCTTTCCAAGGATGTGTGACCGGAATGGTGGTCCGGCGGCTTCGACAGTGGCTGATCGAGCAGATTGA

1 bp insertion

KO (clone 2) TCTGTCTTTCCAAGGATGTGTGACCGGAATGGTGGTACGGCGGCTTCGACAGTGGCTGATCGAGCAGATTGA

1 bp insertion

IRF9

WT CATCAGGCAGGGCACGCTGCACCCGAAAACTCCGGAACTGGGTGGTGGAGCAAGTGGAGAGTG
KO (clone MB5) CATCAGGCAGGGCACGCTGCACCCGAAAACTCC--AACTGGGTGGTGGAGCAAGTGGAGAGTG

2 bp deletion

KO (clone MB5) CATCAGGCAGGGCACGCTGCACCCGAAAAC----AAACTGGGTGGTGGAGCAAGTGGAGAGTG

4 bp deletion
1 bp substitution

IRF7

IRF3/7 WT TGCGCAGCACGCGTCGCTTCGTGATGCTGCGGGATAACTCGGGGGACCCGGCCGACCCGCACAAGGTGTACGCGCTCAGCCGGGAGCTGTGCTG
DKO (clone F10) TGCGCAGCACGCGTCGCTT---TGTACGCCCTCAGCCGGGAGCTGTGCTG

47 bp deletion

IRF3/7 DKO (clone F10) TGCGCAGCACGCGTCGCTTCGTGATGCTGC-------------GGACCCGGCCGACCCGCACAAGGTGTACGCGCTCAGCCGGGAGCTGTGCTG

13 bp deletion

IFNAR2

WT TGGAGAAGCACACACGAGGCCTATGTCACCGTCCTAGAAGGATTC-AGCGGGAACACAACGTTGTTCAGT

KO (clone H17) TGGAGAAGCACACACGAGGCCTATGTCACCGTCCTAGAAGGATTCTAGCGGGAACACAACGTTGTTCAGT
KO (clone H17) TGGAGAAGCACACACGAGGCCTATGTCACCGTCCTAGAAGGATTCAAGCGGGAACACAACGTTGTTCAGT

1 bp insertion

Supplementary Figure 3 legend on the next page.

Supplementary Figure 3: Sanger sequencing of THP1 cell clones to validate IRF and IFNAR2 KOs by CRISPR/Cas9. The gRNA target sequences are colored in green, and the protospacer adjacent motif (PAM) sequences are colored in red. IRF3/7 DKO (clone F10) was generated by first creating an IRF3 KO (clone 4), followed by KO of IRF7.

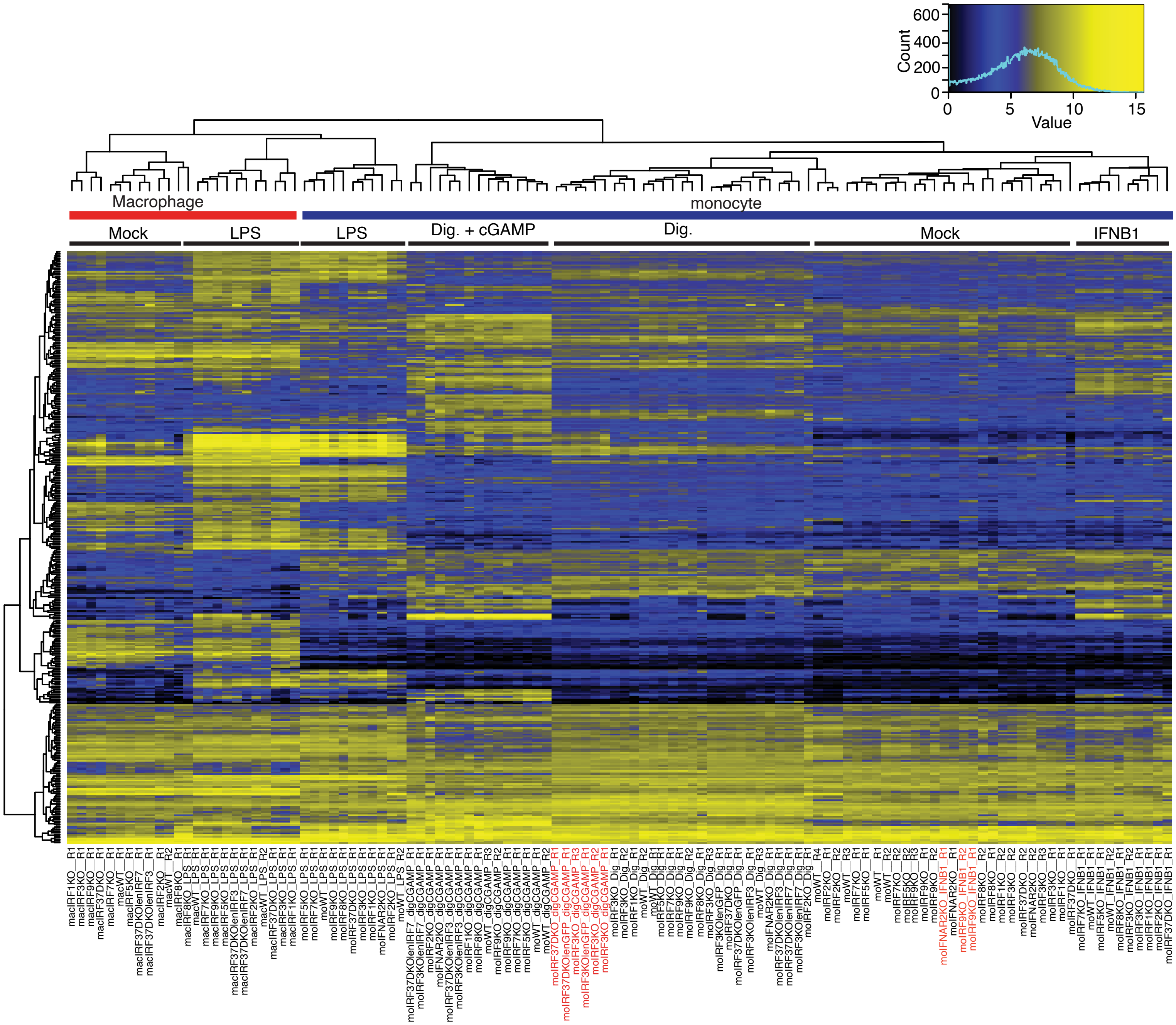

Supplementary Figure 4: Unsupervised clustering of 360 most differentially expressed genes based on pairwise analysis of 114 RNA-seq samples. Refer to the "Methods" section for detailed threshold criteria and cutoff used for selecting the 360 genes. Samples that did not cluster with their expected treatment categories are colored in red.

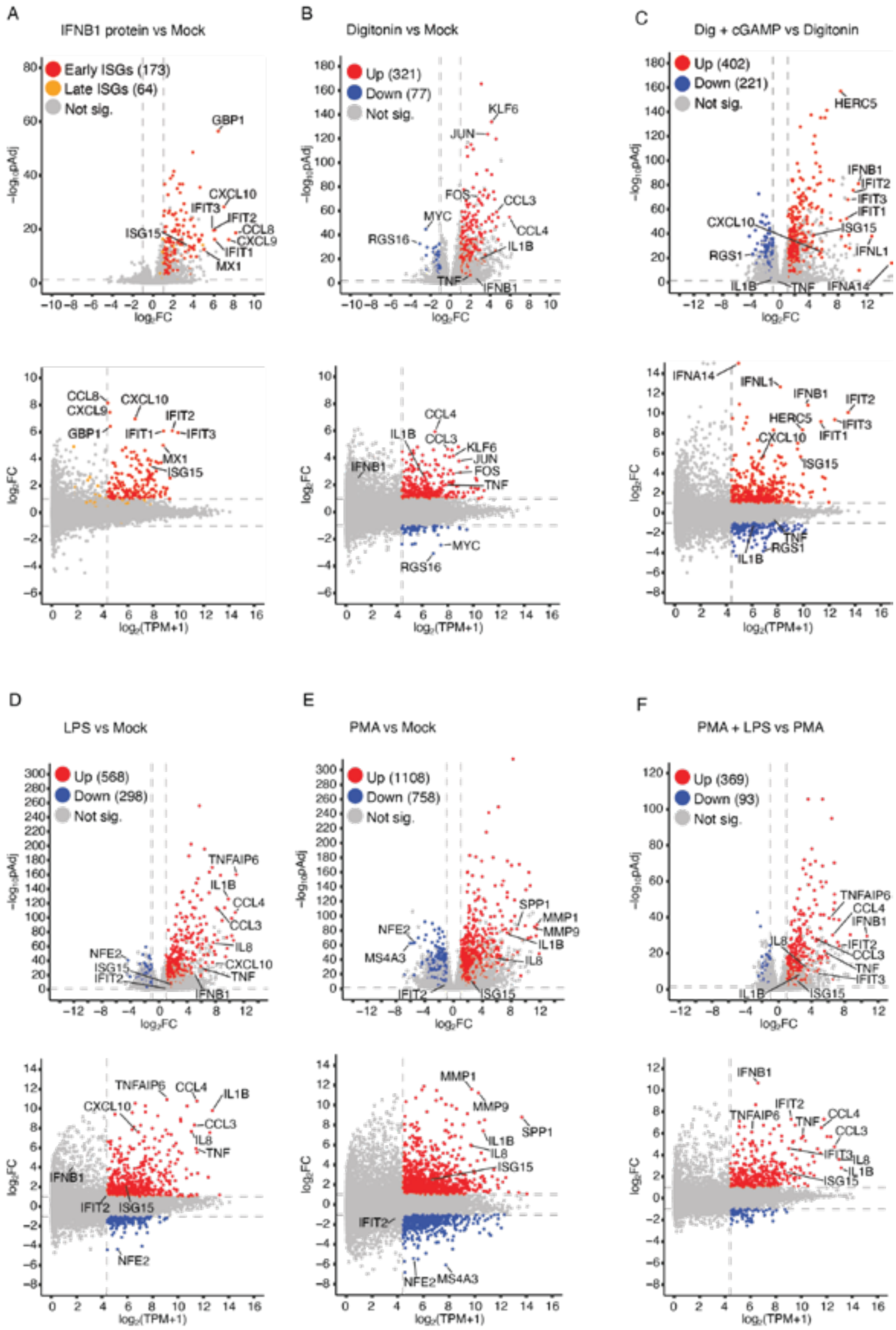

Supplementary Figure 5 legend on the next page.

Supplementary Figure 5: Volcano plot displaying differentially expressed genes across various group comparisons. Significantly regulated genes ($|log_2FC| > 1$ and adjusted p-value ($pAdj < 0.05$)) are shown at the top, while the absolute abundance of the significantly regulated genes is presented at the bottom for each treatment comparison. Labeled genes represent a selection of typical and representative regulated genes.

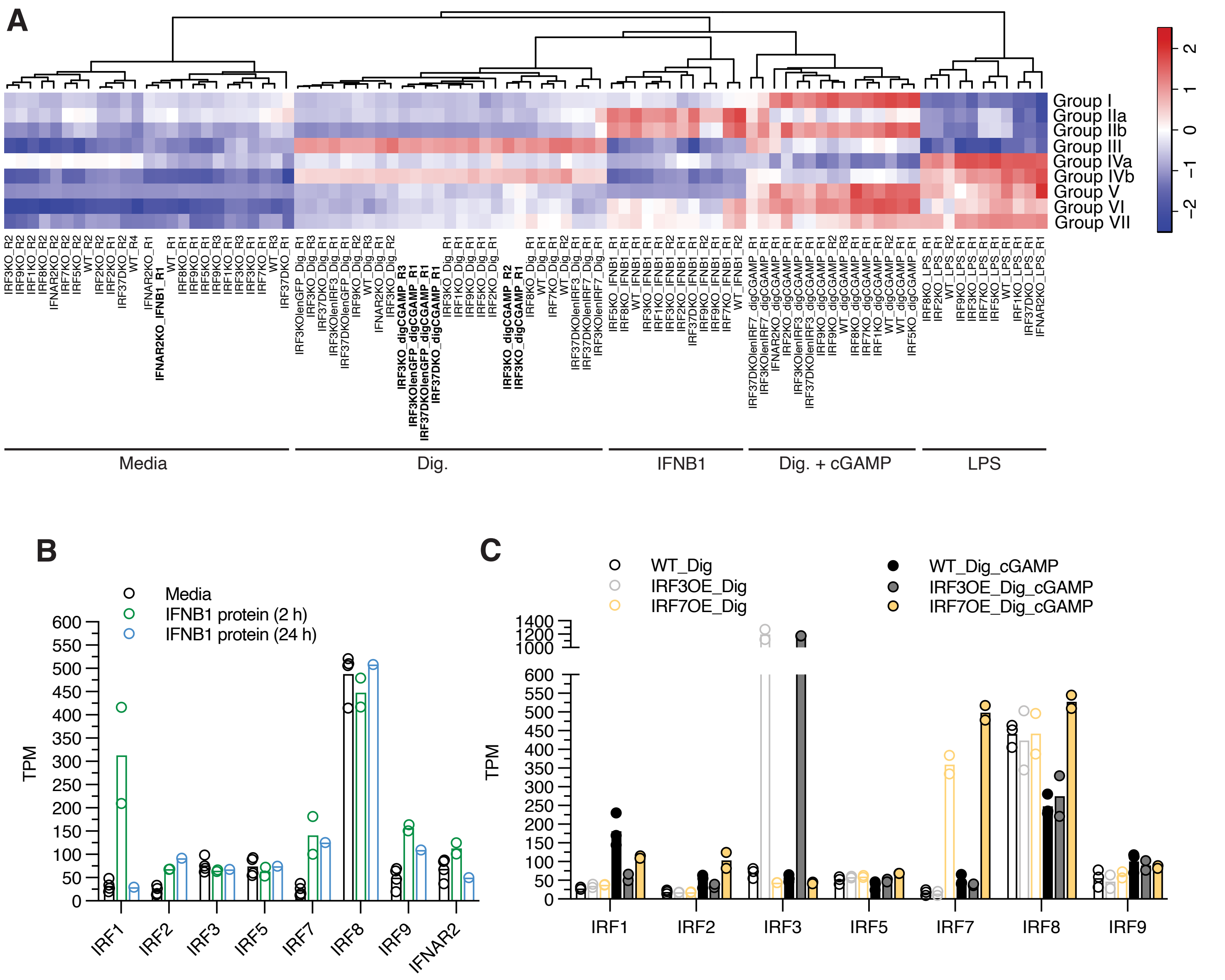

Supplementary Figure 6: A. Unsupervised clustering of averaged z-score values for different gene groups across samples. Sample names for outliers clustering with an unexpected treatment group are displayed slightly lower. Most IRF KOs showed minimal impact on gene induction across various gene groups. **B.** Expression levels (TPM) of IRFs and IFNAR2 in parental THP1 cells following 2 or 24 h IFNB1 protein treatment. **C.** Expression levels (TPM) of IRFs in parental, IRF3 or IRF7 OE THP1 samples. Each data point represents the TPM value for an individual sample, with the mean indicated by the bar, as listed in Supplementary Data 3.

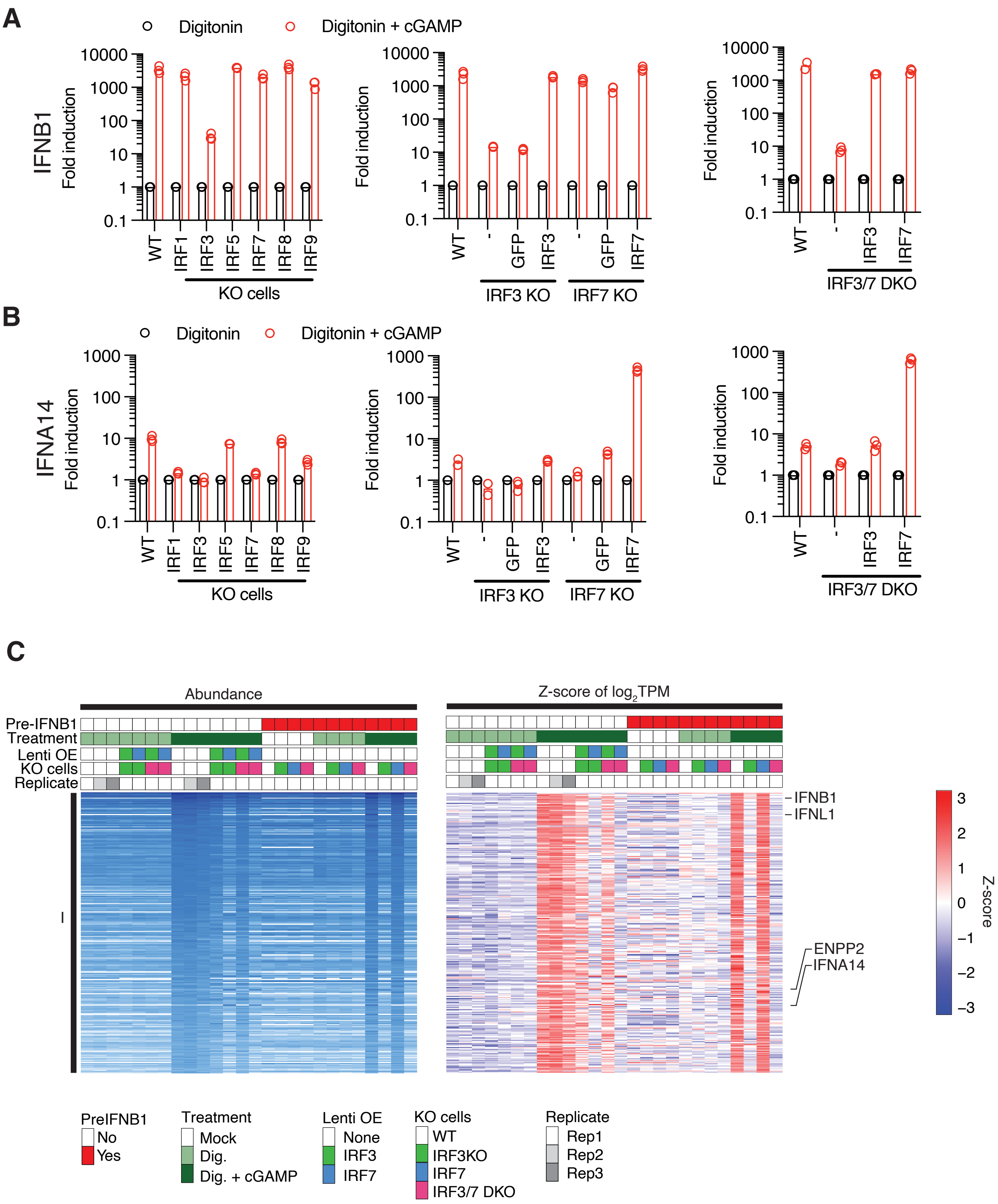

Supplementary Figure 7 legend on the next page.

Supplementary Figure 7: A, B. RT-qPCR analysis of IFNB1 and IFNA14 mRNA expression in various KO cell lines treated with cGAMP. cGAMP induced massive upregulation of IFNB1 mRNA that was dependent solely on IRF3 (**A**, left). Reconstitution of IRF3 or IRF7 restored IFNB1 induction upon cGAMP treatment in IRF3 KO cells (**A**, middle) or IRF3/7 DKO cells (**A**, right). Only IRF7 reintroduction induced IFNA14 mRNA expression (**B**). RT-qPCR data are presented as mean of two or three technical replicates. **C.** Heatmap of Group I genes in IFNB1 protein-pretreated samples for 24 h (preIFNB1) followed by cGAMP exposure for 2 h.

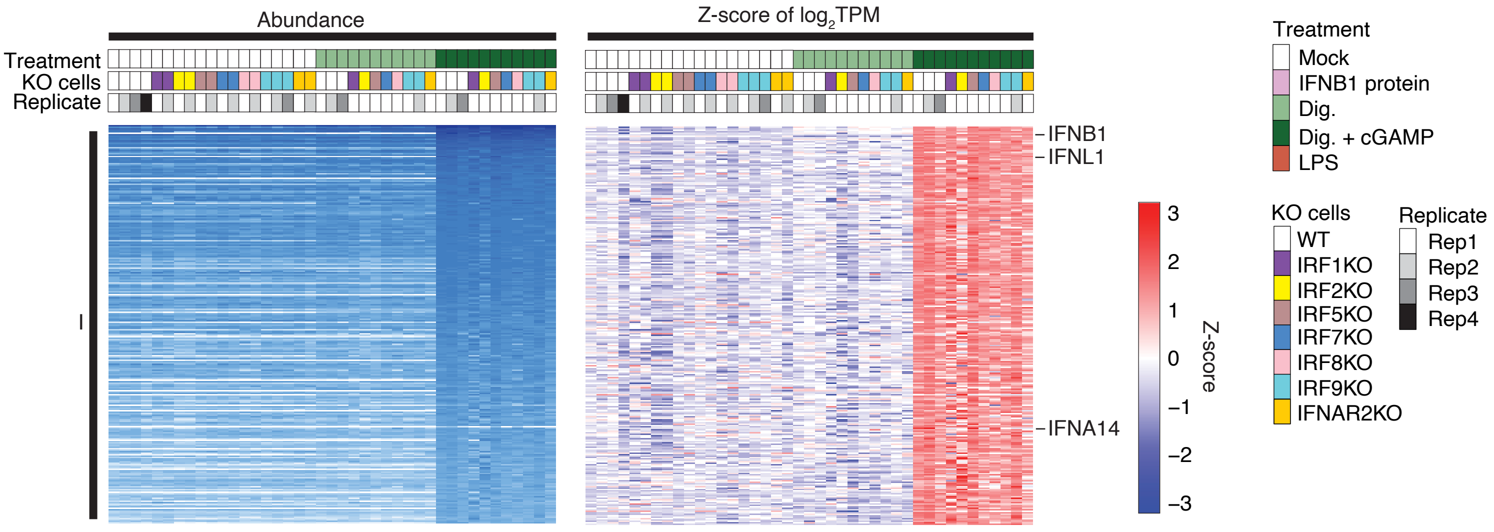

Supplementary Figure 8: Group I genes uniquely induced by cGAMP in wild-type THP1 are unaffected by the absence of IRF1, IRF2, IRF5, IRF7, IRF8, IRF9, or IFNAR2.

A

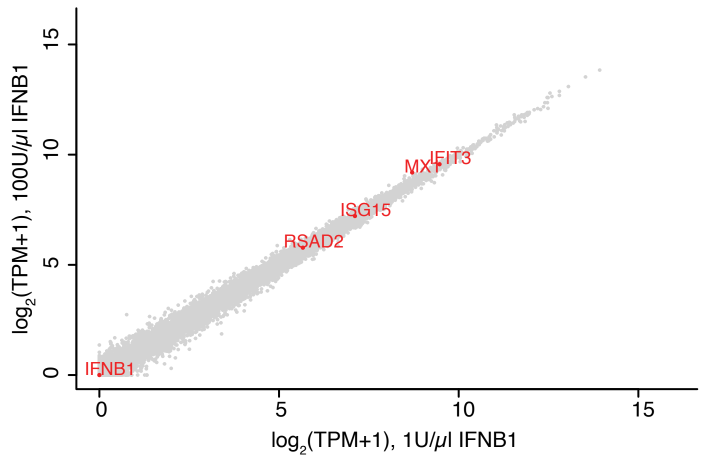

B

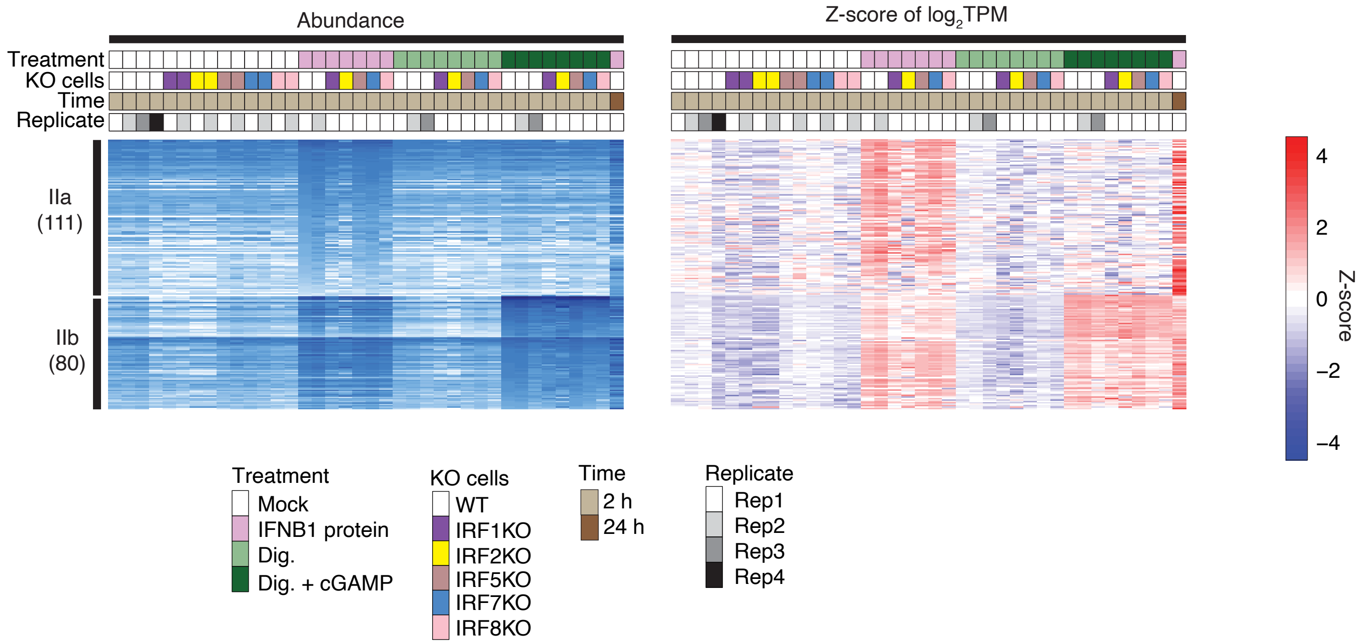

Supplementary Figure 9: A. Scatter plot analysis comparing transcript abundance for THP1 monocytes treated with 1 or 100 U μl^{-1} of IFNB1 protein for 2 h. Representative ISGs and IFNB1 are labeled in red for reference. **B.** Group II (IIa and IIb) genes representing ISGs induced by IFNB1 protein or cGAMP in wild-type THP1 were unaltered by the absence of IRF1, IRF2, IRF5, IRF7, or IRF8.

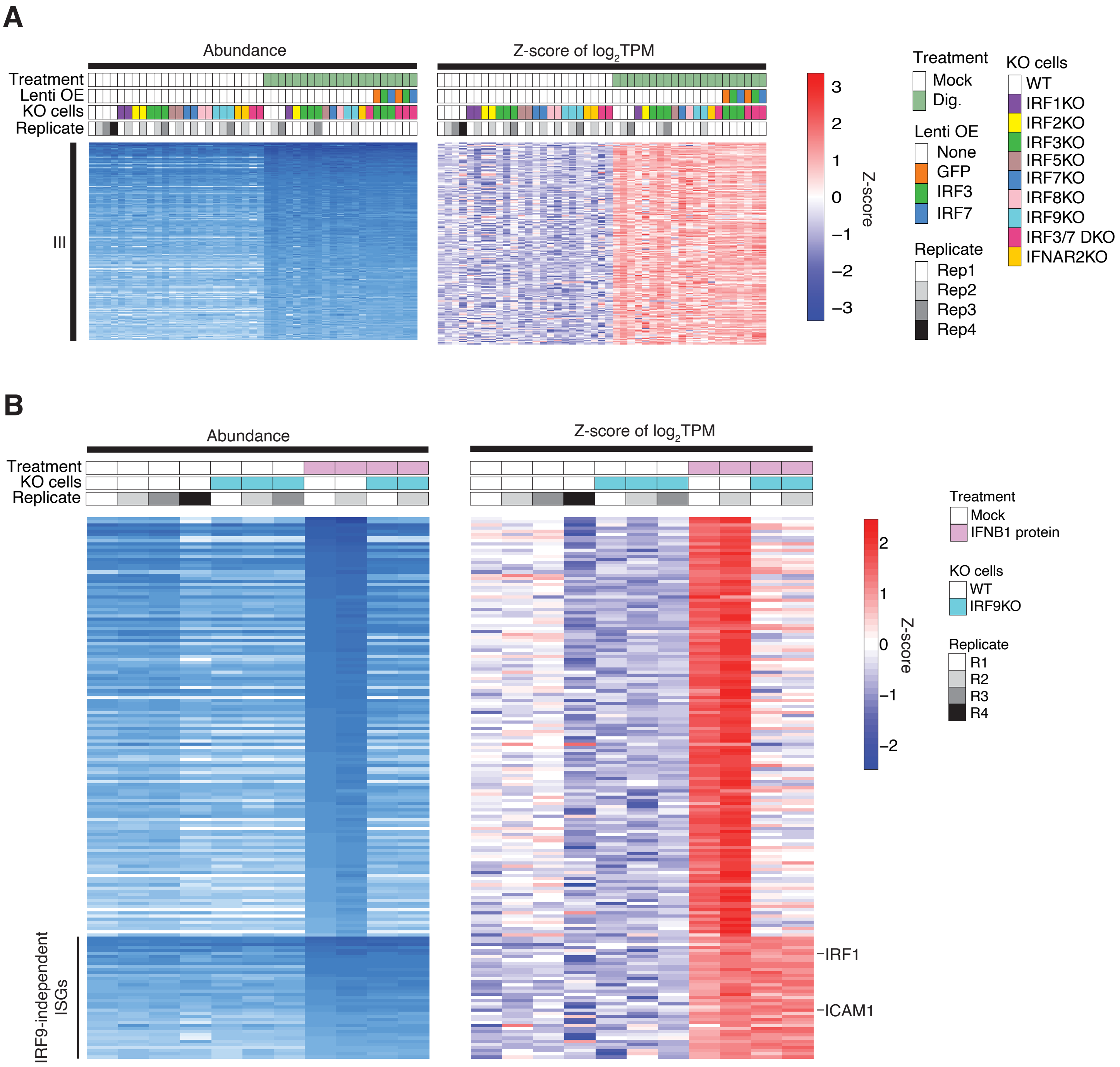

Supplementary Figure 10: A. Group III genes induced by digitonin in THP1 monocytes. Group III genes uniquely induced by digitonin used for membrane permeabilization in wild-type, KO or IRF OE THP1 monocytes. None of the Group III genes were impacted by the absence of the indicated IRFs or IFNAR2. **B.** Identification of IRF9-independent ISGs among 173 ISGs induced after 2 h IFNB1 protein treatment in wild-type THP1 cells using IRF9 KO analysis. The 39 IRF9-independent ISGs are marked with a black side bar. Dig., digitonin; Lenti OE, lentiviral overexpression of the indicated protein.

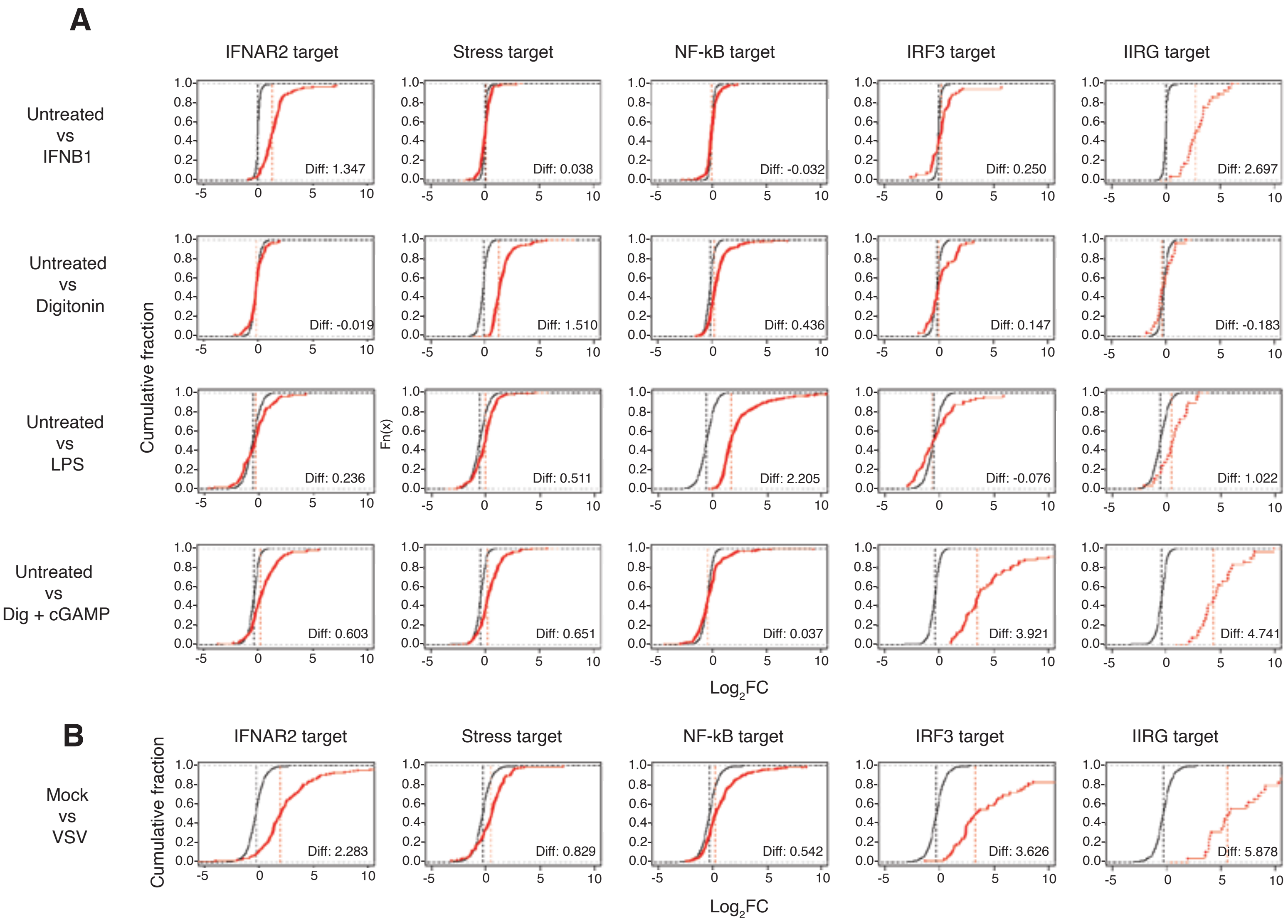

Supplementary Figure 11: A. Cumulative distribution function of expression ratios between untreated and treated wild-type THP1 cells of selected gene sets representing targets of distinct signaling pathways identified in this study (red line) or for ubiquitously expressed control genes (black line). The specific gene sets and corresponding control gene sets used for the analysis are listed in Supplementary Data 5. **B.** The biological relevance of these gene sets was further assessed using THP1 cells infected with Vesicular Stomatitis Virus (VSV), based on a published RNA-seq dataset (GSE199674). Midpoints for each cumulative curve are indicated by dashed vertical lines, and the differences in midpoints (Diff) are shown for each plot.

Supplementary Figure 1B

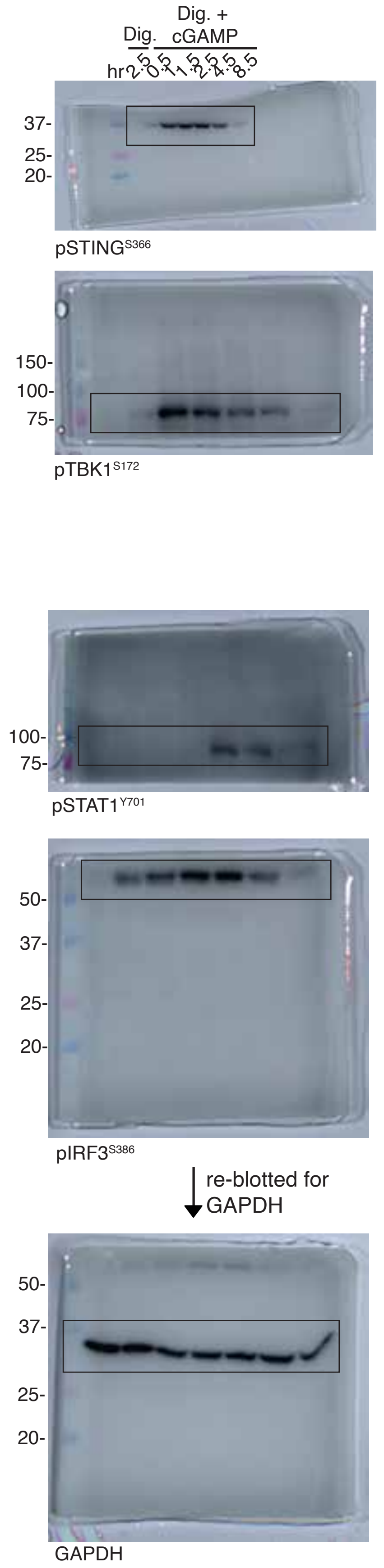

Supplementary Figure 1C

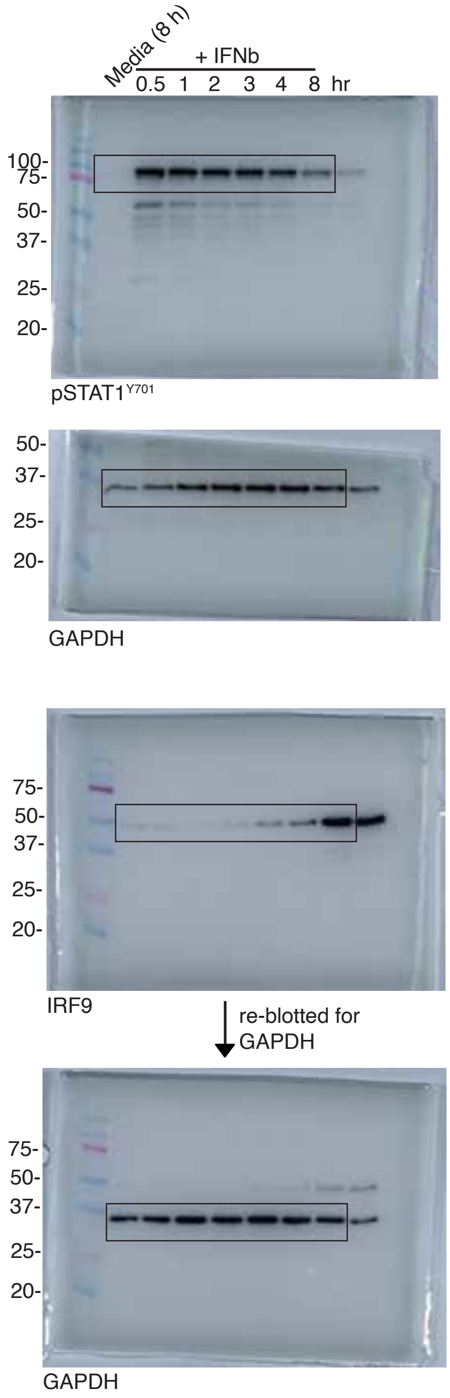

Supplementary Figure 1G

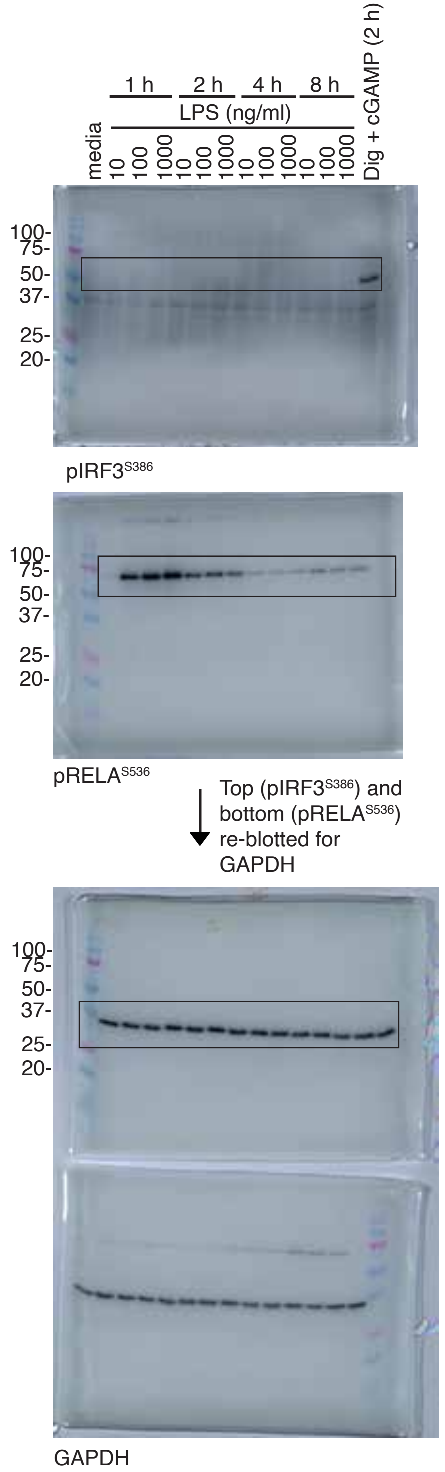

Supplementary Figure 12: Uncropped Western blot images correponding to the the panels shown in Supplementary Figure 1. Rectangular boxes indicate the cropped regions used in the respective figure panels. The antibody applied to each membrane is specified below the corresponding image.

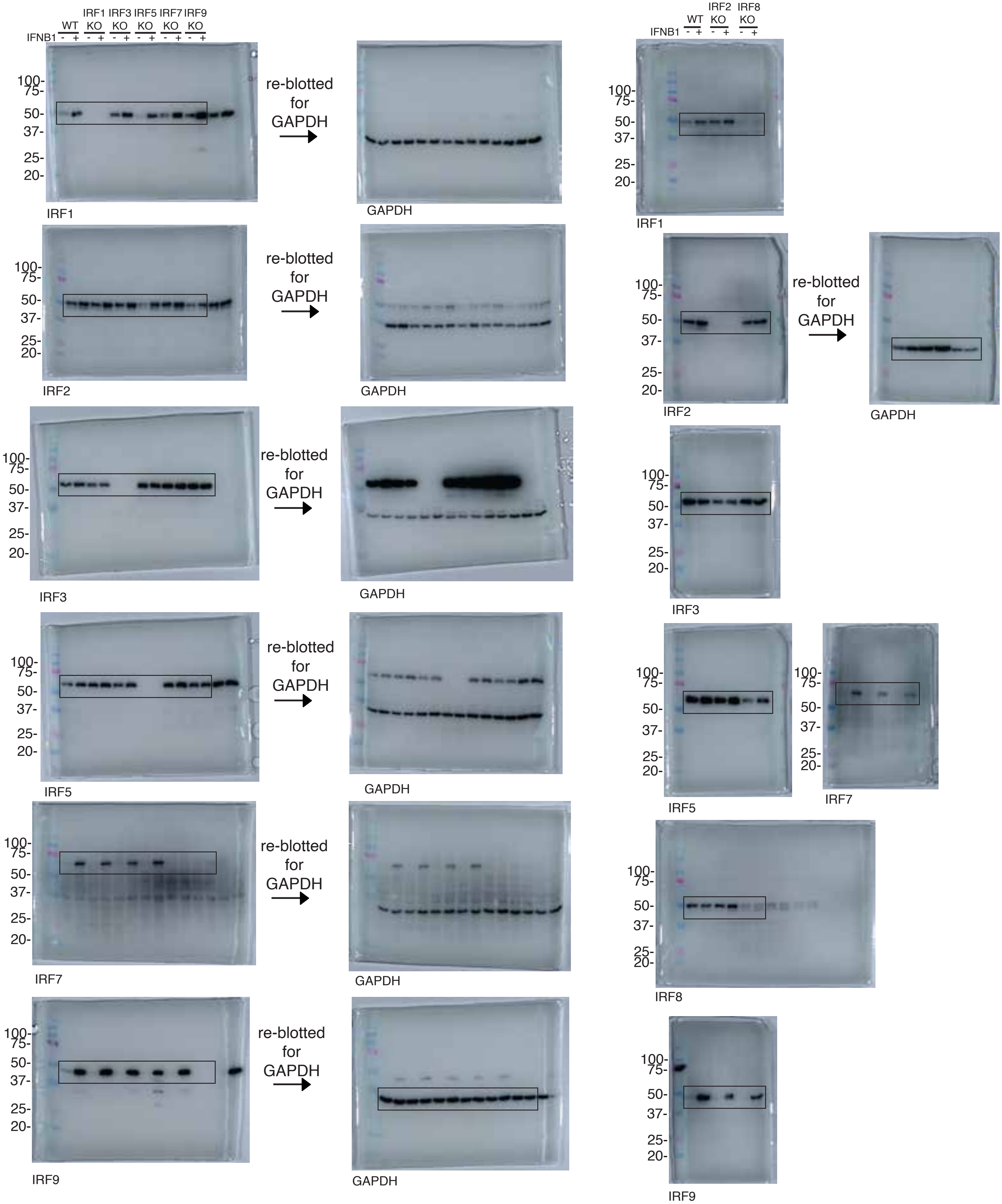

Supplementary Figure 13: Uncropped Western blot images correponding to Supplementary Figure 2A. Rectangular boxes indicate the cropped regions used in the respective figure panels. The antibody applied to each membrane is specified below the corresponding image.

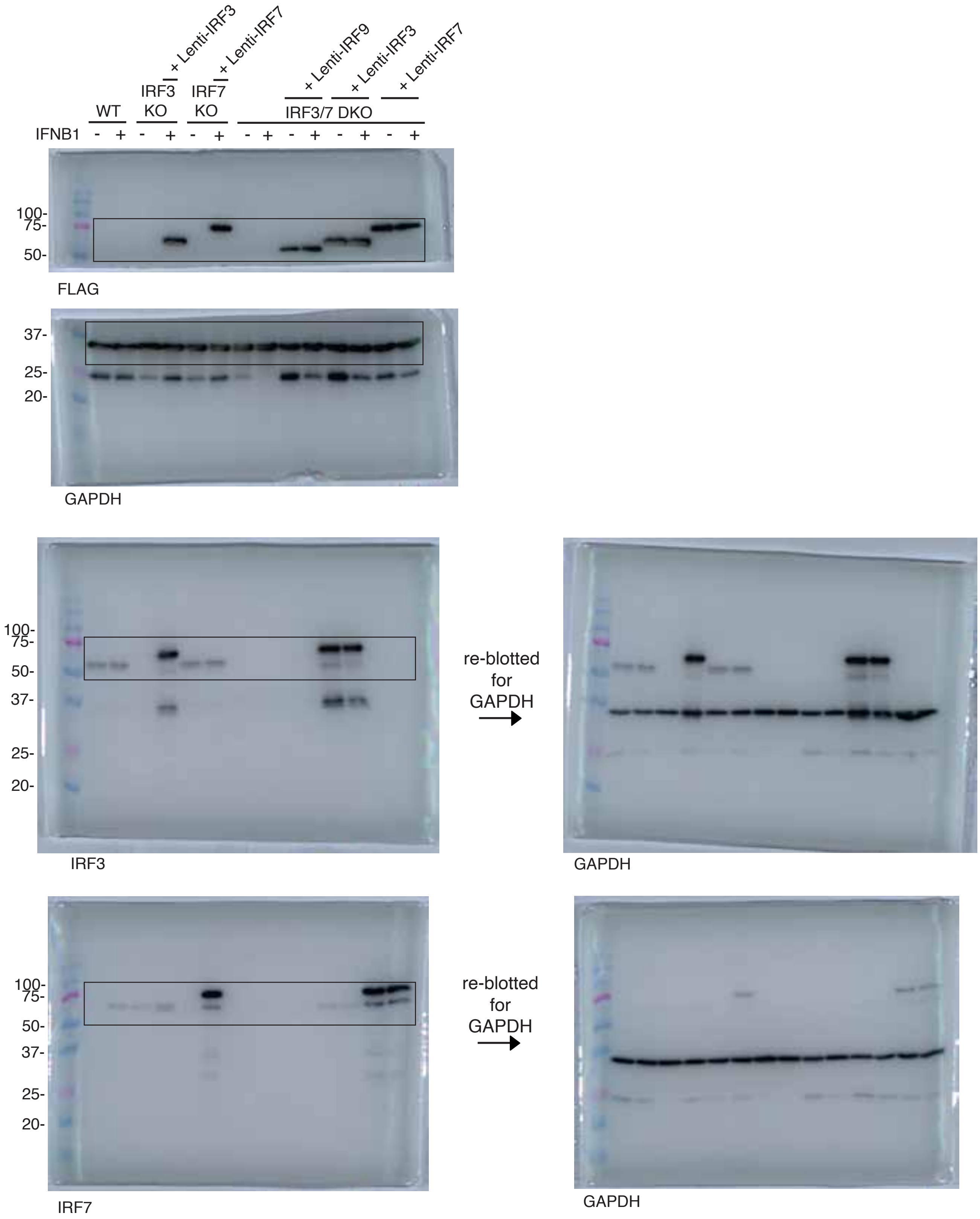

Supplementary Figure 14: Uncropped Western blot images correponding to Supplementary Figure 2B. Rectangular boxes indicate the cropped regions used in the respective figure panels. The antibody applied to each membrane is specified below the corresponding image.

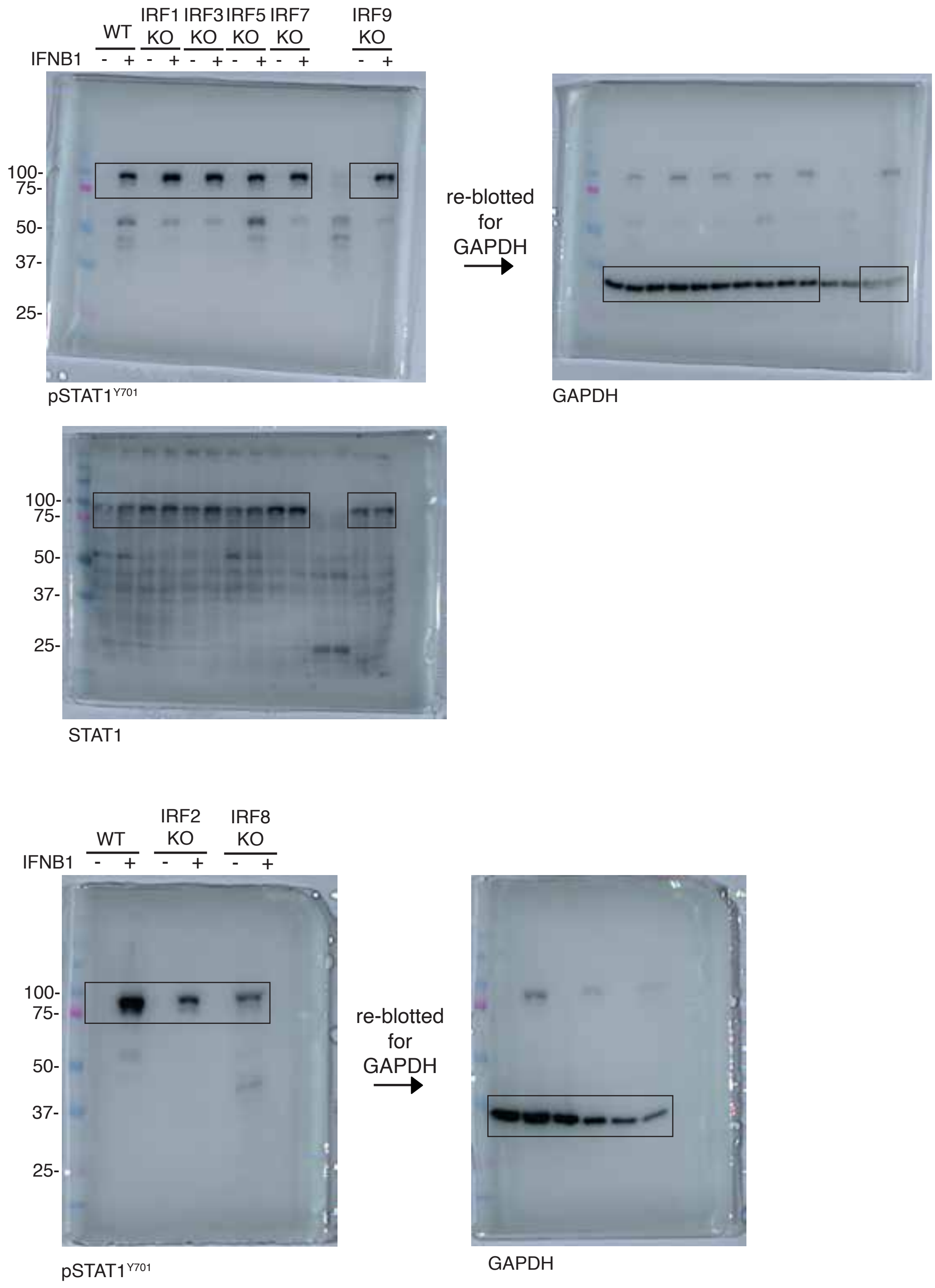

Supplementary Figure 15: Uncropped Western blot images correponding to Supplementary Figure 2C. Rectangular boxes indicate the cropped regions used in the respective figure panels. The antibody applied to each membrane is specified below the corresponding image.

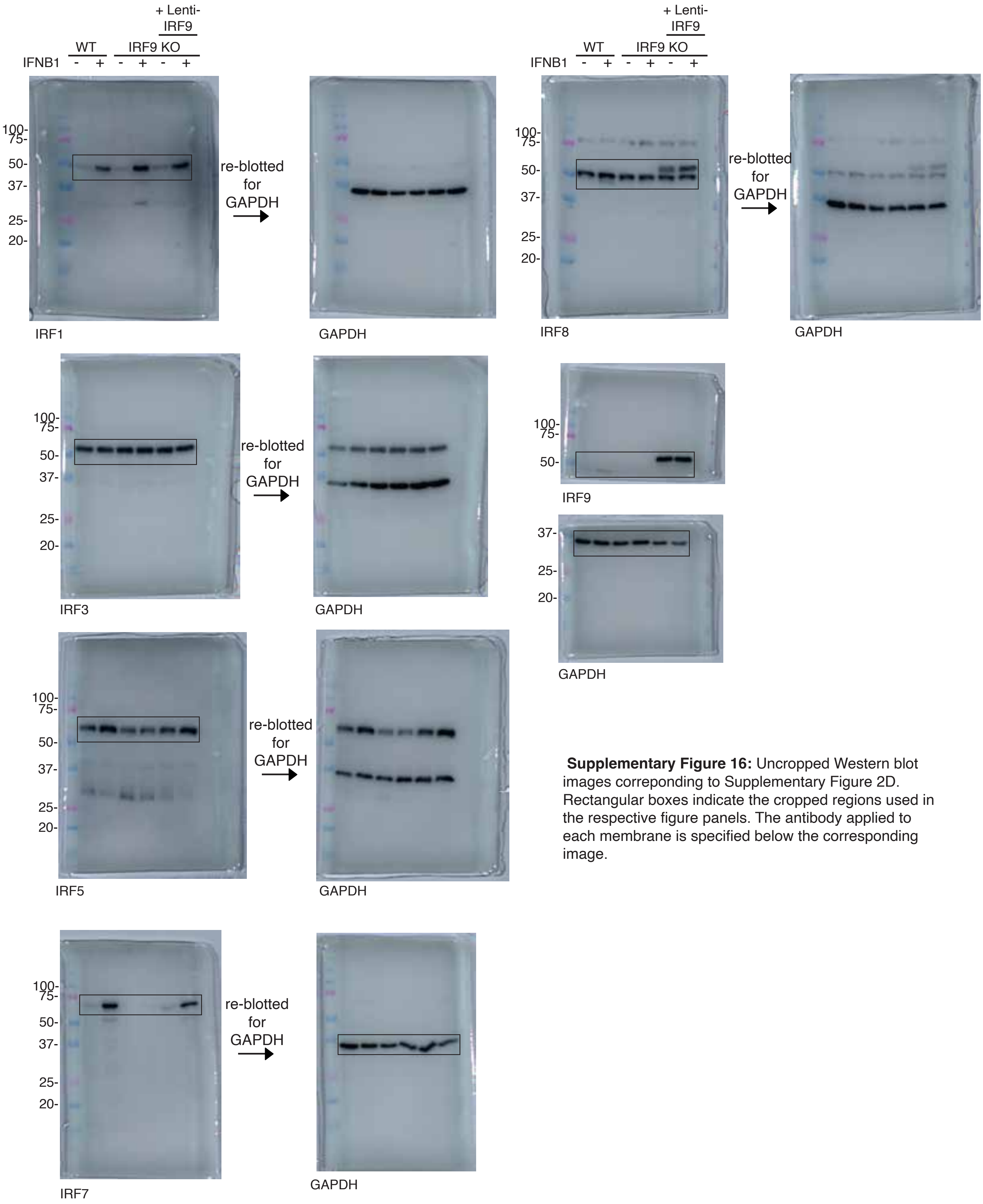

Supplementary Figure 16: Uncropped Western blot images correponding to Supplementary Figure 2D. Rectangular boxes indicate the cropped regions used in the respective figure panels. The antibody applied to each membrane is specified below the corresponding image.

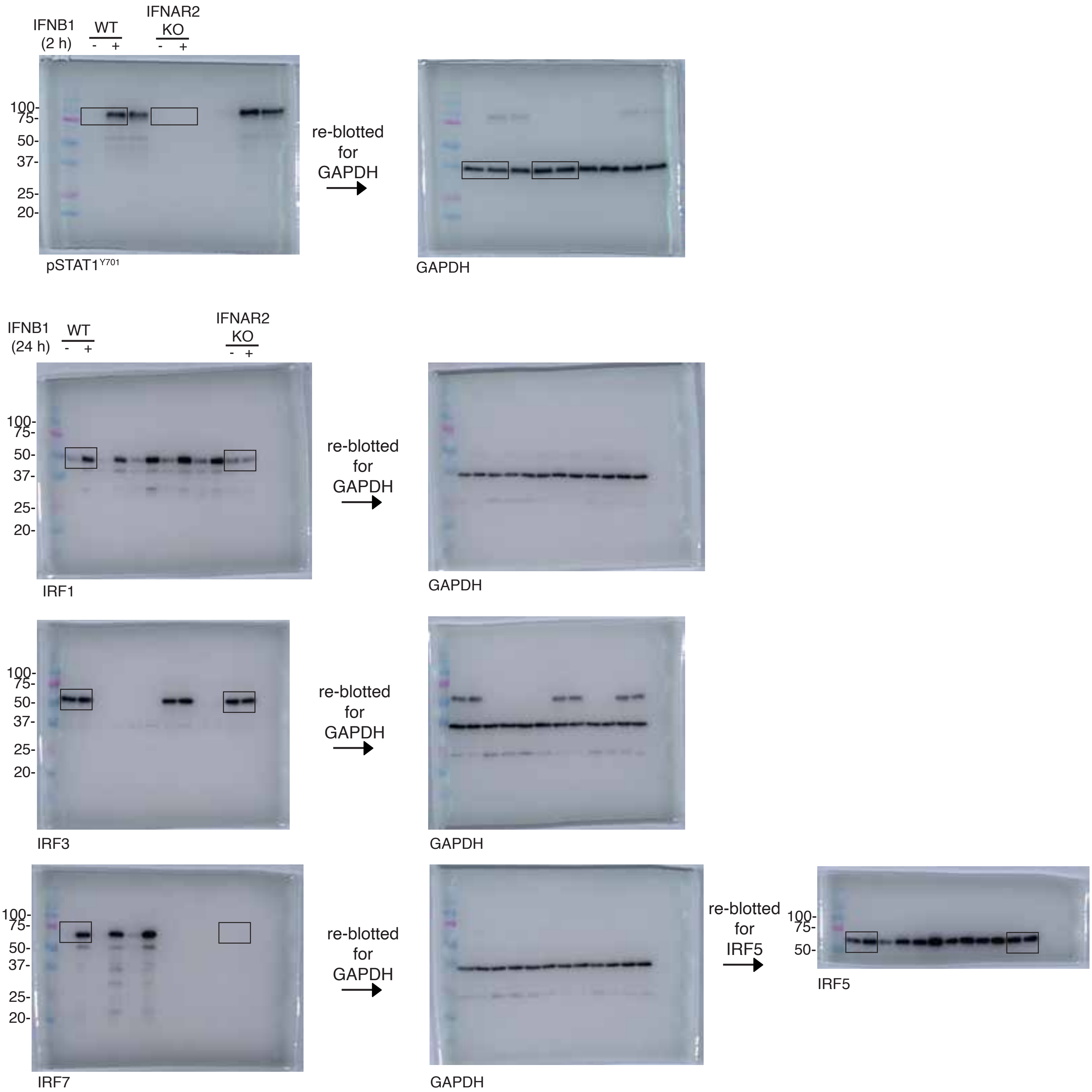

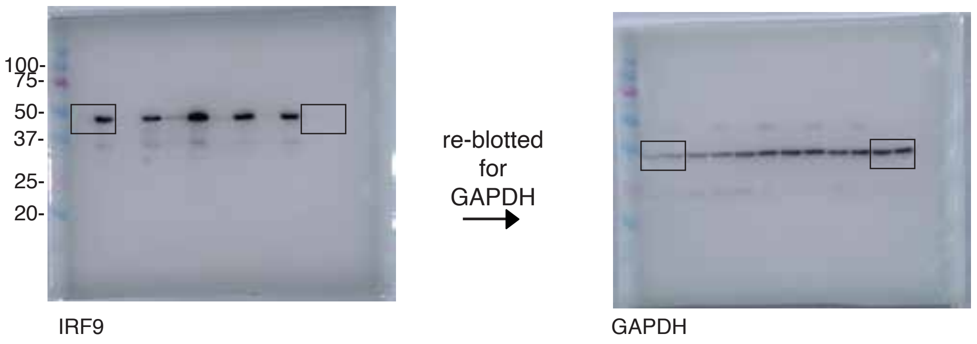

Supplementary Figure 17: Uncropped Western blot images correponding to Supplementary Figure 2E. Rectangular boxes indicate the cropped regions used in the respective figure panels. The antibody applied to each membrane is specified below the corresponding image.

Gene	Species	Forward (5’→3’)	Reverse (5’→3’)
IFNB1	Human	GCTTCTCCACTACAGCTCTTTC	CAGTATTCAAGCCTCCCATTCA
TUBA1B	Human	ACCTTAACCGCCTTATTAGCCA	ACATTCAGGGCTCCATCAAATC
IFNA14	Human	GGCATTGCCCTTTGCTTTAAT	TATTCAGGCTGTGGGTTTGAG
IFIT1	Human	CGCCTGGATGGCTTTAAATTAG	CAGGGCAAGGAGAACCTTAATA

Supplementary Table 1. List of primers used for quantitative RT-qPCR.
