# Supplementary material for: Dissection of innate-immune-ligand- and interferon-protein-mediated transcriptional responses in human THP1 cell states
Source: Commun Biol. 2026 Feb 12;9:239. doi: 10.1038/s42003-025-09343-7 (PMC12901304; doi:10.1038/s42003-025-09343-7)
Supplement: Supplementary file 3 — Reporting Summary [file 42003_2025_9343_MOESM3_ESM.pdf]

nature portfolio

Corresponding author(s): THOMAS TUSCHL

Last updated by author(s): Nov 14, 2025

Reporting Summary

Nature Portfolio wishes to improve the reproducibility of the work that we publish. This form provides structure for consistency and transparency in reporting. For further information on Nature Portfolio policies, see our Editorial Policies and the Editorial Policy Checklist.

Statistics

For all statistical analyses, confirm that the following items are present in the figure legend, table legend, main text, or Methods section.

n/a	Confirmed	
☐	☒	The exact sample size (*n*) for each experimental group/condition, given as a discrete number and unit of measurement
☒	☐	A statement on whether measurements were taken from distinct samples or whether the same sample was measured repeatedly
☒	☐	The statistical test(s) used AND whether they are one- or two-sided<br>*Only common tests should be described solely by name; describe more complex techniques in the Methods section.*
☒	☐	A description of all covariates tested
☒	☐	A description of any assumptions or corrections, such as tests of normality and adjustment for multiple comparisons
☒	☐	A full description of the statistical parameters including central tendency (e.g. means) or other basic estimates (e.g. regression coefficient) AND variation (e.g. standard deviation) or associated estimates of uncertainty (e.g. confidence intervals)
☐	☒	For null hypothesis testing, the test statistic (e.g. *F*, *t*, *r*) with confidence intervals, effect sizes, degrees of freedom and *P* value noted<br>*Give P values as exact values whenever suitable.*
☒	☐	For Bayesian analysis, information on the choice of priors and Markov chain Monte Carlo settings
☒	☐	For hierarchical and complex designs, identification of the appropriate level for tests and full reporting of outcomes
☒	☐	Estimates of effect sizes (e.g. Cohen's *d*, Pearson's *r*), indicating how they were calculated

Our web collection on statistics for biologists contains articles on many of the points above.

Software and code

Policy information about availability of computer code

Data collection	Total RNA for RNA-seq analysis was isolated from each sample using TRIzol (Thermo Fisher) following manufacturer's protocol. Oligo(dT)-selected RNA was converted into cDNA using the Illumina Truseq RNA Sample Preparation Kit v2. Sequencing was performed at The Rockefeller University Genomics Resource Center on the Illumina NextSeq 500 platform with 2x75 nt paired-end.
Data analysis	Annotation was performed using an in-house custom pipeline. Sequencing file quality was assessed with FastQC software (http://www.bioinformatics.babraham.ac.uk/projects/fastqc). Quality-filtered reads were mapped using BOWTIE5, allowing a maximum of two mismatches, against an in-house curated transcript database of the human transcriptome, genome (hg.38). A hierarchical approach was used to assign each read in the order of mRNA, lincRNA, tRNA, miRNA, rRNA, and uncharacterized genomic sequences. Read counts for the mRNA and lincRNA categories were selected for downstream differential gene expression analysis using DESeq2 or normalized to TPM. Data representation and visualization were performed using the R packages ggplot2 and pheatmap.

For manuscripts utilizing custom algorithms or software that are central to the research but not yet described in published literature, software must be made available to editors and reviewers. We strongly encourage code deposition in a community repository (e.g. GitHub). See the Nature Portfolio guidelines for submitting code & software for further information.

Data

Policy information about availability of data

All manuscripts must include a data availability statement. This statement should provide the following information, where applicable:

- Accession codes, unique identifiers, or web links for publicly available datasets
- A description of any restrictions on data availability
- For clinical datasets or third party data, please ensure that the statement adheres to our policy

RNA-seq data are deposited at NCBI Short-Read Archive (SRA) under the BioProject number PRJNA1244336 and are available as of the date of publication. The following public RNA-seq data series were also used in this study: GSE133139, GSE154596, GSE79272, GSE69906, GSE114966, GSE157052, GSE199674, GSE128113, GSE176261 and GSE130011. Expression values for all samples generated, analyzed, and included in this study (Supplementary Data 1-5), and numerical source data for the RT-qPCR plots (Supplementary Data 6), are publicly available in the Dryad repository [DOI: https://doi.org/10.5061/dryad.v15dv428j]. Uncropped blot images are provided in Supplementary Information as Supplementary Figures 12-17.

Research involving human participants, their data, or biological material

Policy information about studies with human participants or human data. See also policy information about sex, gender (identity/presentation), and sexual orientation and race, ethnicity and racism.

Reporting on sex and gender	N/A
Reporting on race, ethnicity, or other socially relevant groupings	N/A
Population characteristics	N/A
Recruitment	N/A
Ethics oversight	N/A

Note that full information on the approval of the study protocol must also be provided in the manuscript.

Field-specific reporting

Please select the one below that is the best fit for your research. If you are not sure, read the appropriate sections before making your selection.

☒ Life sciences ☐ Behavioural & social sciences ☐ Ecological, evolutionary & environmental sciences

For a reference copy of the document with all sections, see nature.com/documents/nr-reporting-summary-flat.pdf

Life sciences study design

All studies must disclose on these points even when the disclosure is negative.

Sample size	RNA-seq was performed on 117 samples that included wild-type and KO cells exposed to different innate stimuli.
Data exclusions	No data exclusion.
Replication	Replicate samples used for the analysis are indicated where available.
Randomization	Sample groups for differential gene expression (DGE) analysis were selected based on pairwise comparisons as detailed in Methods.
Blinding	Not relevant to the study

Reporting for specific materials, systems and methods

We require information from authors about some types of materials, experimental systems and methods used in many studies. Here, indicate whether each material, system or method listed is relevant to your study. If you are not sure if a list item applies to your research, read the appropriate section before selecting a response.

Materials & experimental systems

n/a	Involved in the study
☐	☒ Antibodies
☐	☒ Eukaryotic cell lines
☒	☐ Palaeontology and archaeology
☒	☐ Animals and other organisms
☒	☐ Clinical data
☒	☐ Dual use research of concern
☒	☐ Plants

Methods

n/a	Involved in the study
☒	☐ ChIP-seq
☒	☐ Flow cytometry
☒	☐ MRI-based neuroimaging

Antibodies

Antibodies used

Cell Signaling Technology, IRF1 8478S, IRF7 4920S, IRF8 5628S, IRF9 76684S, STAT1 9175S, pSTAT1-Y701 9167S, pIRF3-S386 37829S, pIRF3-S396 4947S, pTBK1-S172 5483S, pSTING-S366 19781S, NF-kB-RELA 8242S, NF-kB-pRELA-S536 3033S; R&D Systems, IRF2 AF4049; Abcam, IRF3 ab68481, IRF5 ab33478; Proteintech, GAPDH 60004-1-lg; Sigma-Aldrich, FLAG F3165; Dako, polyclonal goat anti-rabbit Immunoglobulins/HRP P0448, polyclonal goat anti-mouse Immunoglobulins/HRP P0447.

Validation

Antibodies were validated using the parental cell line expressing the indicated protein and the knockout cell lines lacking the indicated protein. The knockout cell lines were further verified with Sanger sequencing.

Eukaryotic cell lines

Policy information about cell lines and Sex and Gender in Research

Cell line source(s)

THP1 cells from ATCC TIB-202

Authentication

Cell lines are authenticated by short tandem repeat (STR). For CRISPR-mediated knockout cell lines and lentivirus-mediated knock in cell lines, the presence of genetic alterations were tested at the DNA level by targeted sequencing (Sanger sequencing) and at the protein level by immunoblotting every six months to ensure that the cell identity remains correct and stable throughout the duration of the study.

Mycoplasma contamination

Mycoplasma contamination was routinely checked in all cell lines using e-Myco mycoplasma PCR detection kit (Bulldog Bio).

Commonly misidentified lines
(See ICLAC register)

None.

Plants

Seed stocks

N/A

Novel plant genotypes

N/A

Authentication

N/A
